# Sequential cooperative spectrum sensing in the presence of dynamic Byzantine attack for mobile networks

**DOI:** 10.1371/journal.pone.0199546

**Published:** 2018-07-05

**Authors:** Jun Wu, Tiecheng Song, Yue Yu, Cong Wang, Jing Hu

**Affiliations:** National Mobile Communication Research Lab, School of Information Science and Engineering, Southeast University, Nanjing, China; Beijing University of Posts and Telecommunications, CHINA

## Abstract

Cooperative spectrum sensing (CSS) is envisaged as a powerful approach to improve the utilization of scarce radio spectrum resources, but it is threatened by Byzantine attack. Byzantine attack has been becoming a popular research topic in both academia and industry due to the demanding requirements of security. Extensive research mainly aims at mitigating the negative effect of Byzantine attack on CSS, but with some strong assumptions, such as attackers are in minority or trusted node(s) exist for data fusion, while paying little attention to a mobile scenario. This paper focuses on the issue of designing a general and reliable reference for CSS in a mobile network. Instead of the previously simplified attack, we develop a generic Byzantine attack model from sophisticated behaviors to conduct various attack strategies and derive the condition of which Byzantine attack makes the fusion center (FC) blind. Specifically, we propose a robust sequential CSS (SCSS) against dynamic Byzantine attack. Our proposed method solves the unreliability of the FC by means of delivery-based assessment to check consistency of individual sensing report, and innovatively reuses the sensing information from Byzantines via a novel weight allocation mechanism. Furthermore, trust value (TrV) ranking is exploited to proceed with a sequential test which generates a more accurate decision about the presence of phenomenon with fewer samples. Lastly, we carry out simulations on comparison of existing data fusion technologies and SCSS under dynamic Byzantine attack, and results verify the theoretical analysis and effectiveness of our proposed approach. We also conduct numerical analyses to demonstrate explicit impacts of secondary user (SU) density and mobility on the performance of SCSS.

## Introduction

With the proliferation of wireless services in the last couple of decades, in several countries, most of the available spectrum has fully been allocated, which results in the spectrum scarcity problem. Federal Communications Commission (FCC) research has proved that the main reason for the scarcity of spectrum is the underutilization of the frequency spectrum by the licensed users either temporally or spatially. To improve spectrum utilization and solve the problem of spectrum shortage, cognitive radio (CR) has been widely considered as one of the most promising technologies that can offer a support for the increasing demand for spectrum availability [1]. In cognitive radio networks (CRNs), there are two types of users: primary users (PUs) and secondary users (SUs), and they have different priorities for spectrum access. As an intelligent wireless network framework, SUs equipped with spectrum sensing capability are allowed to opportunistically access the spectrum bands that have been assigned to, but unoccupied by PUs. The SUs are required to evacuate the spectrum bands when PUs become active [2]. Using this access strategy, the spectrum resources can be assured that they can be always in use and thus, spectrum efficiency is enhanced, but without causing interference to PUs.

Spectrum sensing is the key function of CR technology to identify available spectrums. To this end, several spectrum sensing methods have been proposed and investigated in [3] and references therein. However, spectrum sensing techniques do not always guarantee satisfactory performance due to noise uncertainty and channel fading, which are the fundamental characteristics of dynamically changed wireless channel. Once the PU signal experiences deep fading or blocked by obstacles, the power of the PU signal received at the SU may be too weak to be detected [4]. A well-known approach for detecting the PU activity is cooperative spectrum sensing (CSS) where a set of SUs cooperate by fusing their sensing information with each other and collectively deciding on the presence or absence of the PU to enhance the reliability of sensing results.

But the nature of aggregating data makes CSS open a window for attackers to sneak into collaborative SUs, who will send out falsified local spectrum inference to the fusion center (FC). During such an assault, attackers can prevent reliable SUs from using the existing white space, or allure them to access the channels in use and cause excessive interference to PUs [5]. This typical sort of attack in CSS is Byzantine attack, which is also referred to as spectrum sensing data falsification (SSDF), in pursuit of the CSS performance degradation, thereby undermining the premise of CR technology.

Byzantine attack in CSS systems is a critical threat and has attracted considerable attention recently. Chen R et al. in [6] presented a hybrid method called weighted sequential probability ratio test (WSPRT). Their method combines the nodes’ reputation and uses sequential probability ratio test (SPRT) to identify attackers. The WSPRT was also investigated in [7] for a centralized CRN and a novel fusion scheme based on spatial correlation technique was proposed. The authors utilize geographical and reputational weights to define a two-level FC for secure collaborative sensing. On the basis of WSPRT, [8] analyzed the CSS performance when there exist attackers based on the cumulative reputation, and proposed an effective scheme by employing Orthogonalized Gnanadesikan-Kettenring (OGK) to improve the robustness of CRNs. In [9], Sharifi AA and Niya JM proposed an attack-aware CSS (ACSS) method to estimate the credit value of each CR user and identify attackers along with their attack strategies. The similar work was presented in [10], an attack-aware WSPRT algorithm estimates the attack extension factor in a network, based on the standard deviation of received sensing reports for improving the CSS performance. In contrast to existing data fusion techniques that use a fixed number of samples, WSPRT algorithm uses a variable number of samples with only the cost of a relatively low overhead. The authors of [6]–[10], however, assume an ideal FC but pay little attention to the problem of the FC being compromised.

In another work, the authors focused on a new kind of attack model which is called balanced collaborative (BC) attack in [11]. With the assistance of trusted nodes, an abnormality detection algorithm was proposed to detect BC attackers based on the theoretical analysis that the reports between BC attackers have the highest similarities. Likewise, [12] proposed a simple yet robust secure CSS scheme with hard decision combining for opportunistic spectrum access (OSA) networks. Based on the historical sensing records and the assistance of trusted OSA nodes, the proposed approach effectively excludes misbehaving OSA nodes from the process of cooperation. And [13] presented a new weighted likelihood ratio rest (WLRT) to mitigate the effect of Byzantine attack, in which the collaborative weight is calculated by comparing the sensing history of each user with the reliable anchor node’s global decision. However, all of them invariably take advantage of reliable helper nodes or anchor nodes to identify attackers or falsified reports, such an assumption is not realistic in a real network.

In order to effectively defend against SSDF attack behaviors from malicious SUs, [14] proposed a faithworthy CSS scheme based on the Dempster-Shafer (D-S) theory, including four consecutive procedures, which are basic probability assignment (BPA) with the projection approximation approach, holistic credibility calculation, option and amelioration for BPA and evidence combination via the D-S rule, respectively. Besides D-S theory that is criticized to have high decision conflicts, some common methods (clustering-based, consensus theory, etc.) are widely used in the field of prevention of Byzantine attack, which only function well in some specific attacks because of convergence limitation. [15] presented prevention and trust evaluation scheme, called IRTrust, the framework of which incorporates a strategy of identity authentication and a global trust of peers defend against several kinds of malicious attacks, such as simple malicious attacks, collusive attacks, strategic attacks, and sybil attacks. Building trusted relationships among peers in a large-scale distributed peer-to-peer (P2P) system is of particular concern to L. Li et al, rather than a centralized one.

Moreover, existing algorithms fail to take the mobility of SUs into consideration. To address this problem, considering the location diversity of the network, [16] divided the whole area of interest into several cells as well as [17]. Each user’s reputation score is updated after each sensing slot and is used for identifying whether it is malicious or not. If so, it would be removed away. And then the proposed reputation-based cooperative spectrum sensing (RCSS) algorithm assigns users in cells with better channel conditions, i.e. larger signal-to-noise ratios (SNRs), with larger weighting coefficients, without requiring the prior information of SNR. Pollution attack similar to Byzantine attack has been recently investigated by [18] [19].

Although the researchers worldwide have made significant progress in addressing the research challenges associated with CSS, robust CSS remains a big challenge for the science and engineering community. The above-mentioned CSS methods against Byzantine attack are invariably based on a simple attack model. From the attacker’s point of view, “always attack” is a radical attack strategy, but is not necessarily the optimal, especially in the presence of countermeasures. Despite that extensive simulation results prove the outstanding performance in attacker identification, the premise that the number of attackers is less than that of reliable SUs is indispensable. Therefore, the problem of Byzantine attack making the FC blind is largely ignored. Besides, for those who are identified by the network as attackers, the common practice of these methods is that the FC arbitrarily eliminate them from the process of CSS upon discovery. These issues motivate us to address the question:

- If the FC’s global decision is no longer reliable, is there any other way to accurately measure the local sensing report, in addition to the auxiliary node?
- What is the relationship between the attacker ratio and attack probability, especially when the FC is blind?
- In view of CSS, is the attacker’s sensing information regarding the primary signal definitely useless information?

The study of these practically motivated questions requires in-depth analyses, which wil be systematically considered in our work.

Starting from the network model, the performance study of existing spectrum sensing algorithms often overlooks the impact of SU mobility. Many of them assume SUs stationary or with low mobility. As an addition to the wireless communication technology CR system should consider mobility in spectrum [20], which is followed by spectrum sensing model and Byzantine attack model.

Prior work on Byzantine attack mainly concentrates on strategies that work in small regions where a common ground truth is viable, and attackers constitute a small fraction of the secondary’s or use unsophisticated strategies. Such limitations fuel the motivation in providing a more generic attack model by intensively observing malicious behaviors, from a sophisticated attacker’s viewpoint, to conduct more flexible attack strategies. Under the generalized model, we analyze the circumstance where Byzantine attack makes the FC blind and derive a closed form expression of the blind condition.

In the front of Byzantine attack, two kinds of data fusion techniques, voting rule and hypothesis test, are introduced and elaborated from different angles, such as, the CSS performance, the ability of resisting attack and the number of samples required. Moreover, we make a detailed analysis on WSPRT and its drawbacks.

Then comes the last and most important, a sequential CSS (SCSS) is proposed by a structured and comprehensive overview of research on Byzantine attack, which consists of delivery-based assessment, reusing weight allocation, trust value (TrV) ranking-based sequential test. In the proposed SCSS, delivery-based assessment supersedes the FC’s global decision to check consistency of received local reports in case Byzantine attack makes the FC blind, but without extra cost. Also, we consider that the falsified sensing report submitting to the FC contains some useful information, and can be distinguished and utilized by observing malicious behaviors instead of arbitrarily eliminating. This is the main idea of reusing weight allocation. At last, we integrate the presence and absence decision ability into the weight of each report, and proceed with sequential test in TrV ranking order.

The remainder of this paper is organized as follows. In Section II, system model is formulated, including network model and Byzantine attack model. Section III gives theoretical analyses on the scenario where Byzantine attack makes the FC blind. Section IV reviews current data fusion techniques including voting rule and hypothesis test. A robust SCSS is proposed in Section V to defend against dynamic Byzantine attack. Finally, simulation results are provided in Section VI to verify our proposed method, and conclusions are drawn in Section VII.

## System model

### Network model

Considering Byzantine attack has more impact in a centralized network wherein false information can propagate quickly [21]. The system model is a centralized CRN consisting of one primary transmitter (regarded as PU in the CRN), one FC and *N* collaborative SUs in which coexists *N*_*a*_ attackers, where *ρ* = *N*_*a*_/*N*. In fact, our proposed method also can be applied in the network with several PUs and FCs.

#### Mobile scenario

Most of the work related to spectrum sensing assume that the SUs are stationary. However, mobility is an essential feature of wireless networks [22]. As such, it is critical to consider spectrum sensing with mobile users in the network.

**Fig 1:**
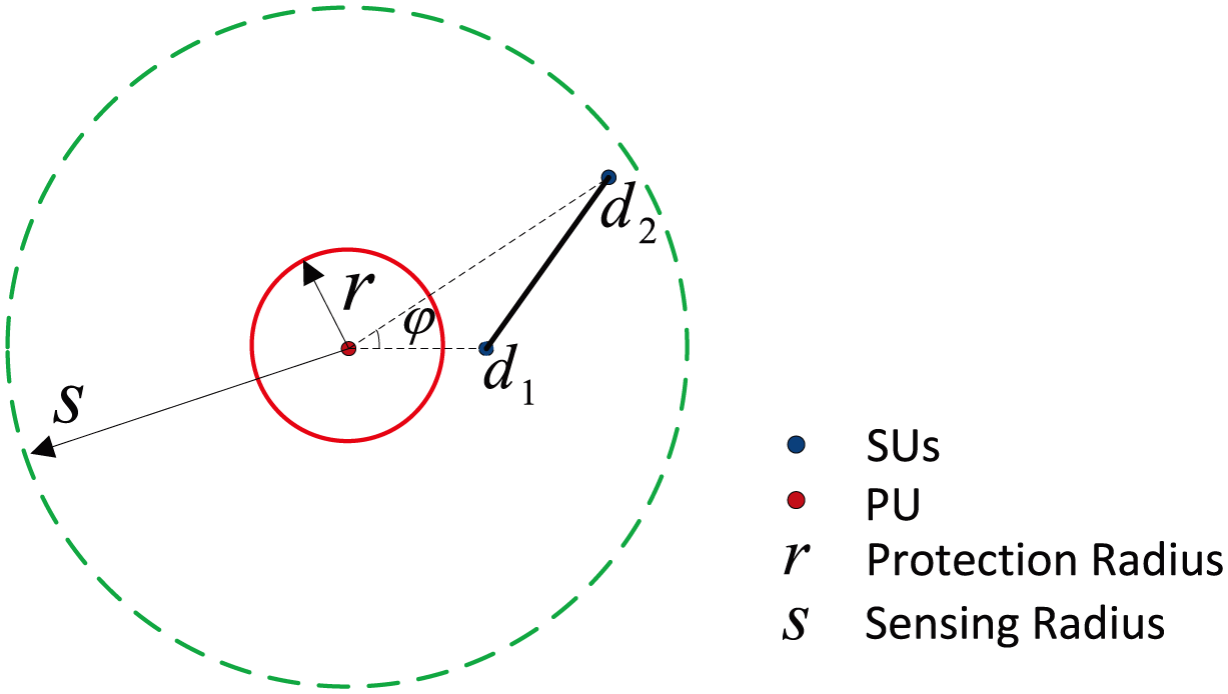
Illustration of mobile scenario.

We present a system model to imitate a scenario where the PU is stationary and the SUs are mobile in the random waypoint model (RWM) in Fig 1. The PU is located at the center of the network. At the beginning of the epoch, the SU chooses a new waypoint *d*_1_ toward the destination waypoint *d*_2_. There is no pause time between two waypoints. Each SU has an identical sensing range (sensing radius is *s*) to sense the licensed channel. To protect the PU’s effective communication, SUs are forbidden to access licensed channel within the protection range (protection radius is *r*) of PU.

#### Local spectrum sensing

In order to opportunistically access available spectrums, common spectrum sensing methods include matched filter, energy detection, cyclostationary detection, and wavelet detection can be adopted, among which energy detection is the most widely used because of its low complexity and it does not require any prior knowledge about the primary signal. In the energy detection, the PU signal detection can be formulated as a binary hypothesis test problem as follows:

**Hypothesis** *H*_1_

Due to the SU mobility, the relative position of SUs and the PU varies over time, according to the log-normal shadowing path loss model, the received PU power at a distance *d* can be expressed as in dB [23]:

$$\begin{array}{ccc} P_{r}\left(dB\right) & = & P_{t}-PL\left(d\right)=P_{t}-\bar{PL}\left(d\right)-\psi \\ & = & P_{t}-\bar{PL}\left(d_{0}\right)+10llog_{10}\left(\frac{d}{d_{0}}\right)+\psi \end{array}$$ (1)

where *PL*(*d*) is the path loss as a function of *d* and $\bar{PL}\left(d\right)$ is the mean of *PL*(*d*), *d*_0_ is a close-in reference distance which is determined from measurements close to the transmitter of PU. *l* is the path loss exponent which indicates the rate at which the path loss increases with distance, and *ψ* is a Gauss-distributed random variable with mean zero and variance *σ*^2^.

Under hypothesis *H*_1_, the a priori probabilities can be computed as

$$\begin{array}{ccc} P(s_{i}=1|H_{1}) & = & P\left(s_{i}>\gamma |H_{1}\right)=P\left(P_{t}-\bar{PL}\left(d\right)-\psi >\gamma \right)\\ & = & Q(\frac{\gamma -P_{t}+\bar{PL}\left(d\right)}{\sigma }) \end{array}$$ (2)

and

$$\begin{array}{ccc} P(s_{i}=0|H_{1}) & = & 1-P(s_{i}=1|H_{1})\\ & = & Q(\frac{P_{t}-\bar{PL}\left(d\right)-\gamma }{\sigma }) \end{array}$$ (3)

where *Q*(⋅) is the complementary distribution function of the standard Gaussian, and *γ* is the pre-determined detection threshold.

**Hypothesis** *H*_0_

$$\begin{array}{c} P_{r}=n_{0} \end{array}$$ (4)

where *n*_0_ can be regarded as a Gaussian noise power with mean *n*_0_ and standard deviation *σ*_*n*_.

Under hypothesis *H*_0_, the a priori probabilities can be computed as

$$\begin{array}{c} P\left(s_{i}=1|H_{1}\right)=Q\left(\frac{\gamma -n_{0}}{\sigma_{n}}\right) \end{array}$$ (5)

and

$$\begin{array}{c} P\left(s_{i}=0|H_{1}\right)=Q\left(\frac{n_{0}-\gamma }{\sigma_{n}}\right) \end{array}$$ (6)

It is worth noting that each SU submits raw signal power measurement or a bit decision to the FC according to soft/hard-combining technology, but the binary hard-combining is more advantageous since there is no need for a powerful FC which results in reduced costs, but our results can be readily extended to the case of soft-combining. Some methods of quantifying sensing data have been investigated in [24] [25], including quantized hard-combining; however, this study is beyond the scope of our work.

### Dynamic Byzantine attack model

Considering the importance of not causing interference to the primary network, spectrum sensing, as a fundamental problem in CRNs, requires SUs to efficiently and effectively detect the presence of the PU, but SUs’ changeable environment and ease of compromise, such as shadowing and multipath fading, lead to the fact that the local spectrum sensing conducted by the individual user is often incorrect. CSS is suggested to improve the detection accuracy. In CSS, SUs sense the medium individually and share their local report results to find a consensus on the channel availability. SUs send their local report results to the FC that aggregates all SUs reports and makes the global decision. Assume that the communication channels between SUs and the FC are error-free in this paper.

**Fig 2:**
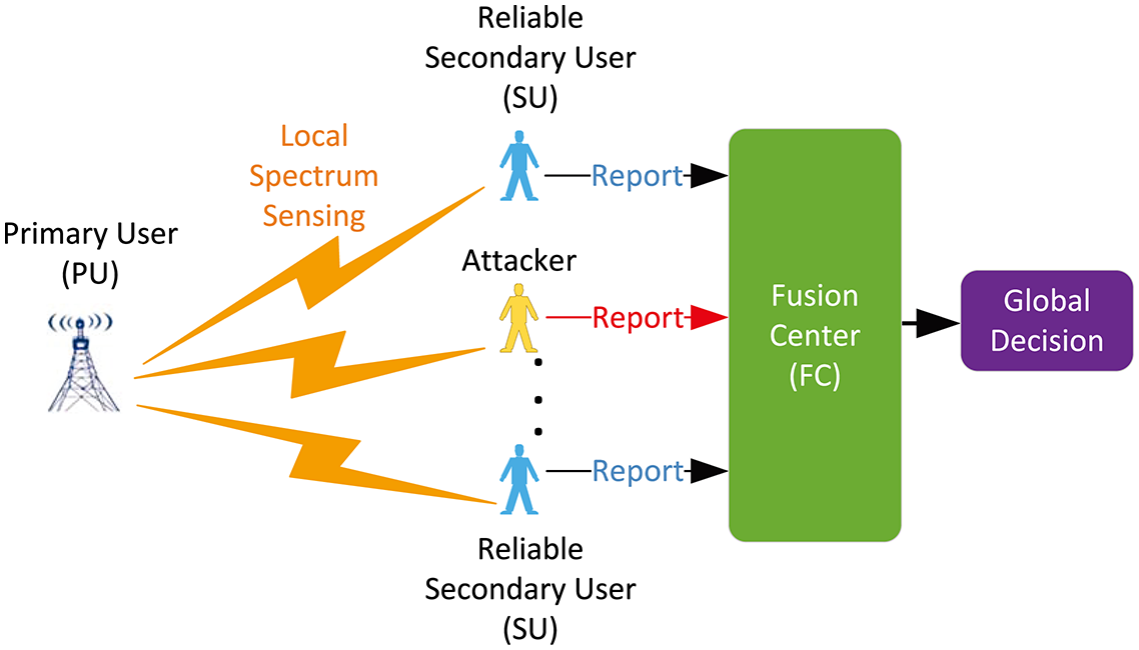
Cooperative spectrum sensing model in the presence of Byzantine attack.

CSS can successfully mitigate the disadvantages of local spectrum sensing. However, it also poses a great challenge in terms of Byzantine attack, since it allows attackers to take advantage of the cooperative paradigm [26]. Byzantine attack is represented by attackers that send false sensing results to the FC, trying to mislead the global decision regarding the spectrum occupancy, as shown in Fig 2.

By concretely analyzing a general area of related work to Byzantine attack, we make a summary of attack type in accordance with the way that an attacker sends the false sensing report as follows:

- The first type of attack is the one who always declares that the PU is active, called Always-Yes (AY) attack. Via AY attack, attackers can access to the idle channel exclusively at the expense of other SUs.
- The second type of attack is the one who always reports an absence of primary signal, called Always-No (AN) attack, thereby imposing severe interference on the PU.
- The third type is the one who always reports the opposite of what the attacker has sensed, called Always-False (AF) attack, which has the negative impacts of both AY and AN attack.

Undoubtedly, “always attack” is a simple yet special case of Byzantine attack. As an attacker who considers the attack risk or attack cost, “always attack” may not be the optimal strategy. If an attacker always reports the false information to the FC, such a static attack strategy can be easily identified. In order to perform stealth attack, the attacker is bound to adjust the attack strategy in consideration of changeable environment and countermeasures.

**Fig 3:**
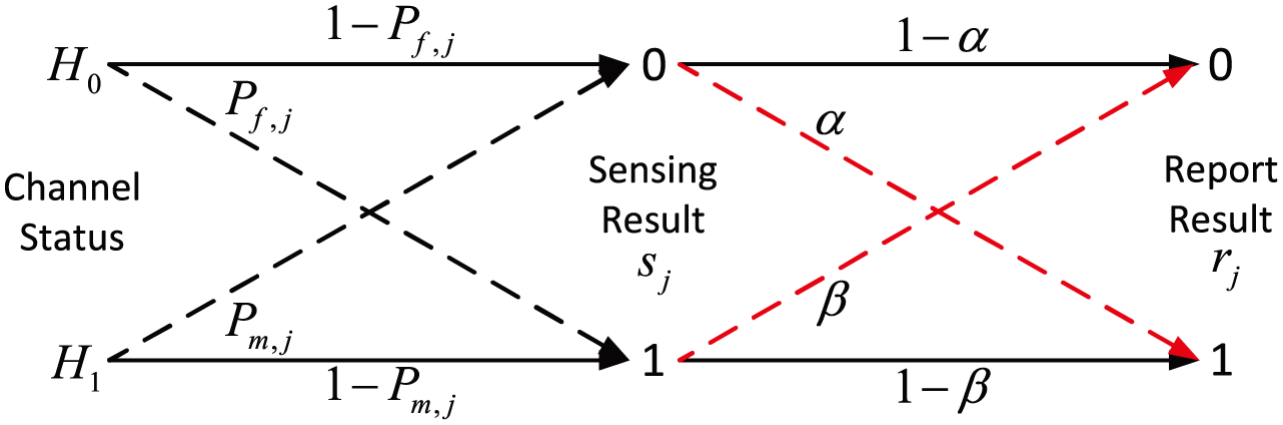
Dynamic Byzantine attack model.

On this account, the attack probability will be appropriately set by the attacker [27], in pursuit of sneaking into a reliable SU. Here, we propose a dynamic Byzantine attack model, as shown in Fig 3., including false alarm attack (FAA) and miss detection attack (MDA), which is described as

$$\begin{array}{c} \left\{ \begin{array}{c} P(r_{j}=1|\left(s_{j}=0|H_{0}\right))=\alpha \\ P(r_{j}=0|\left(s_{j}=1|H_{1}\right))=\beta \end{array}\right. \end{array}$$ (7)

where *s*_*j*_ is the sensing result and *r*_*j*_ is the report result, *α* is the FAA probability, *β* is the MDA probability.

Depending on the above dynamic attack model, obviously, AY attack can be regarded as a special FAA with the attack probability is 1 (*α* = 1), while AN attack as a special MDA the attack probability is 1 (*β* = 1). AF attack boils down to a combination of AY and AN attack (*α* = *β* = 1). Furthermore, as for attack strategy, *α* = *β* is too ideal to be achieved in [9] [28] etc. Such a view totally overlooks the fact that FAA and MDA are independent. Thus, according to the attack probability, the false alarm and miss detection probability of the *j*-th attacker can be represented as

$$\begin{array}{c} P_{fa,j}=P\left(r_{j}=1|H_{0}\right)+P\left(r_{j}=1|H_{0}\right)=P_{f,j}\left(1-\beta \right)+\left(1-P_{f,j}\right)\alpha \end{array}$$ (8)

and

$$\begin{array}{c} P_{ma,j}=P\left(r_{j}=0|H_{1}\right)+P\left(r_{j}=0|H_{1}\right)=P_{m,j}\left(1-\alpha \right)+\left(1-P_{m,j}\right)\beta \end{array}$$ (9)

where *P*_*da*,*j*_ = 1 − *P*_*ma*,*j*_ is the detection probability.

## Byzantine sophistication

Under the generalized Byzantine attack model, attackers have a certain probability, varying from 0 to 1, to conduct various attacks. In the practical network, attackers could adopt sophisticated and destructive strategies rather than a simple one to undermine the network operability (the FC’s ability of making the correct decision regarding the presence or absence of the PU [29]). If possible, they would want to make the FC completely unable to decide on a particular decision, i.e., to make the performance of CSS no better than a random guess of the channel status [30]. To secure the process of CSS, we further investigate such a scenario in this section.

We say that the FC is blind if attackers can make the sensing report that the FC receives from the SUs, such that no information is conveyed. In other words, the FC cannot perform better than simply making the decision based on priors [31]. Most of the previously conducted research usually accounted for alleviating the effect of Byzantine attack when the percentage of attackers is relatively small. Whereas little research has been done regarding the scenario where the attacker makes the FC blind. This deficiency motivates us to address the question: what is the condition of which Byzantine attack makes the FC blind? In the Bayesian framework, the blind implies that the report result received by the FC is completely independent of the hypothesis test, that is, the blind condition can be stated as

$$\begin{array}{c} P\left(r|H_{0}\right)=P\left(r|H_{1}\right) \end{array}$$ (10)

where **r** = [*r*_1_, *r*_2_, …, *r*_*N*_] is the report vector received by the FC.

Considering that each SU’s sensing obseravtion is subject to conditional indepentdent and identically distribution. The miss detection and false alarm probability are assumed to be the same for every SU irrespective of whether they are reliable or not, denoted by *P*_*m*_ and *P*_*f*_, respectively. Therefore, the blind condition of (10) can be rewrited as

$$\begin{array}{c} \rho \left(\alpha \left(P_{f}+P_{m}-1\right)+\left(1-\beta \right)\left(1-P_{f}-P_{m}\right)\right)+\left(1-\rho \right)\left(1-P_{f}-P_{m}\right)=0 \end{array}$$ (11)

Consequently,

$$\begin{array}{c} \rho =\frac{1}{\alpha +\beta } \end{array}$$ (12)

When *α* = *β* = 1 (AF attack), a critical value of 50% attackers can completely blind the FC. Additionally, for *α* = 1, *β* = 0 (AY attack), only 100% attackers can lead to the blind result and the same attack ratio is required for *α* = 0, *β* = 1 (AN attack). Another interesting extension is that the attack probability and the attack ratio can be compensated for each other when the blind condition is satisfied.

## Overview of data fusion techniques

As illustrated in Fig 2, Byzantine attack makes CSS lie in a tremendous risk, the fusion technology for the local sensing information received by the FC is particularly critical to guarantee the integrity of CRNs. Existing data fusion technologies toward spectrum sensing generally fall into voting rule and hypothesis test. In this section, a brief description of them are presented, which is followed by a presentation of their advantages and disadvantages in detail.

### Voting rule

Voting rule (a.k.a. counting rule or *K*-out-of-*N* rule) is one of the simplest data fusion technologies. It is available for making the global decision and contains three kinds of widely adopted rule, such as, Or, And, Majority rule.

Or rule is a simple decision rule described as that no less than one reports the presence of the PU, then the FC broadcasts the channel is busy, i.e., *H*_1_ is accepted if $\sum_{i}^{N}r_{i}\geq 1$, otherwise *H*_0_. And rule works as if all decisions report the presence of the PU, then the FC broadcasts the channel is busy, i.e., *H*_1_ is accept iff $\sum_{i}^{N}r_{i}=N$, otherwise *H*_0_ is accepted. Majority rule is based on the majority of the individual decisions. If more than half of decisions report the presence of the PU, then the FC broadcasts the channel is busy, i.e., *H*_1_ is accepted if $\sum_{i}^{N}r_{i}=N\geq \left\lceil N/2\right\rceil$, otherwise *H*_0_ is accepted.

As is clear from the above description, both the detection and false alarm probability of And rule are very low, while Or rule are the opposite. Or, And, or Majority rule can be generalized as voting rule. In a generalized voting rule, *K* is usually used as an optimization variable to obtain the optimal system performance, i.e. [28]. In summary, voting rule can be realized in low complexity without any prior knowledge on the PU signal. But no matter what kind of voting rules is adopted, a fixed number of samples is required to inefficiently make the global decision.

### Hypothesis test

Similar to voting rule, hypothesis test is performed to test the binary decision on the presence of the PU in CRNs. There are different test methods adopting various design rules [27], such as, Bayesian detection, Neyman-Pearson (N-P) test, and SPRT. All of hypothesis tests require varying degrees of prior information for the global decision output.

#### Bayesian detection

Bayesian detection requires the knowledge of a priori probabilities of hypotheses *H*_*θ*_(*θ* = 0, 1), denoted by *P*(*H*_*θ*_), and the conditional probabilities *P*(*r*_*i*_|*H*_*θ*_). Thus, four possible decision cases can occur in the binary hypothesis test problem [32], two correspond to correct decisions including *r*_*i*_ = 1 when the PU is active and *r*_*i*_ = 0 when the PU is inactive, and the other two to errors including *r*_*i*_ = 0 when the PU is active and *r*_*i*_ = 1 when the PU is inactive. The objective of Bayesian detection is to minimize the average detection cost given by

$$\begin{array}{c} C_{ave}=\sum_{\vartheta =0}^{1}\sum_{\theta =0}^{1}C_{\vartheta \theta }P\left(r_{i}=H_{\vartheta }|H_{\theta }\right)P\left(H_{\theta }\right) \end{array}$$ (13)

where *C*_*ϑθ*_ represents the cost of declaring *H*_*ϑ*_ true when *H*_*θ*_ is present. Accordingly, likelihood ratio test (LRT) of Bayesian detection is described as

$$\begin{array}{c} \prod_{i=1}^{N}\frac{P(r_{i}|H_{1})}{P(r_{i}|H_{0})}{}_{\overset{<}{H_{0}}}^{\overset{H_{1}}{>}}\;\frac{P\left(H_{0}\right)\left(C_{10}-C_{00}\right)}{P\left(H_{1}\right)\left(C_{01}-C_{11}\right)} \end{array}$$ (14)

The right side of (14) is equivalent to the threshold of Bayesian decision *λ*_*Bayes*_.

#### N-P test

Unlike Bayesian detection, neither the prior probabilities on hypotheses or detection costs associated with decision cases are prerequisite in N-P test, but the conditional probabilities *P*(*r*_*i*_|*H*_*θ*_) are also indispensable. The objective of N-P test is to design a nonrandomized test that maximizes the detection probability while guarantees the false alarm probability to be lower than an acceptable value [27]. The N-P test based LRT is represented as

$$\begin{array}{c} \prod_{i=1}^{N}\frac{P(r_{i}|H_{1})}{P(r_{i}|H_{0})}{}_{\overset{<}{H_{0}}}^{\overset{H_{1}}{>}}\;\lambda_{NP} \end{array}$$ (15)

where *λ*_*NP*_ is the detection threshold derived from the acceptable false alarm probability.

As (14) and (15) show, Bayesian detection and N-P test are both essentially a fixed-number LRT; their only difference is the way that the threshold is chosen [6].

#### SPRT

The main advantage of sequential test is that it requires, on an average, fewer samples to achieve the same probability of error performance as a fixed sample size test [32]. In SPRT, samples sequentially arrive at the FC, the test statistic iteratively proceeds until a final decision is made. Define the following LRT as

$$\begin{array}{c} S_{l}=\prod_{i=1}^{l}\frac{P(r_{i}|H_{1})}{P(r_{i}|H_{0})} \end{array}$$ (16)

where the number of samples *l* varies from 1 to *N*. *S*_*l*_ is compared with a lower threshold *λ*_*l*_ and an upper threshold *λ*_*u*_ to make a final decision, the test procedure is described as follows [6]

$$\begin{array}{c} \left\{ \begin{array}{c} S_{l}\geq \lambda_{u},\;\text{accept}\;H_{1}\\ S_{l}\leq \lambda_{l},\;\text{accept}\;H_{0}\\ \lambda_{l}<S_{l}<\lambda_{u},\;\text{take}\;\text{another}\;\text{observation} \end{array}\right. \end{array}$$ (17)

The values of double thresholds *λ*_*l*_ and *λ*_*u*_ are defined as

$$\begin{array}{c} \lambda_{l}=\frac{B}{1-A},\lambda_{u}=\frac{1-B}{A} \end{array}$$ (18)

where *A* and *B* are the tolerated false alarm and miss alarm probability, respectively.

### WSPRT

When applying SPRT to data fusion for CSS, fewer samples can be in charge of making the global decision about the primary signal. Nevertheless, SPRT is incapable of identifying falsified data in the presence of Byzantine attack, therefore, Chen R et al. proposed WSRPT to remedy the shortcoming by a dynamic weight in [6]. Before proceeding with the description of our CSS scheme, a brief of the classic WSPRT against Byzantine attack is provided in this subsection.

#### Review of WSPRT

In WSPRT, two steps are implemented by weight allocation and sequential test. In the weight allocation, a SU’s TrV is related to its detection accuracy. If a SU’s local report is consistent with the global decision, its TrV will be increased; otherwise it will be decreased. Then, the *i*-th SU’s TrV at the *k*-th sensing interval is updated as

$$\begin{array}{c} T_{i}\left(k\right)=T_{i}\left(k-1\right)+{\left(-1\right)}^{\left(r_{i}\left(k\right)+d\left(k\right)\right)} \end{array}$$ (19)

where *d*(*k*) represents the global decision from the FC. Consequently, integrating the SU’s TrV into the weight, a SU with a larger TrV has a larger weight. Using a normalized function, the weight allocation mechanism can be followed as:

$$\begin{array}{c} w_{i}\left(k\right)=f\left(T_{i}\left(k\right)\right)=\left\{ \begin{array}{c} 0,T_{i}\left(k\right)\leq -g\\ \frac{T_{i}\left(k\right)+g}{max(T_{i}\left(k\right))+g},T_{i}\left(k\right)>-g \end{array}\right. \end{array}$$ (20)

where *g* = 5. More detailed considerations about weight allocation mechanism of (20) refers to [6].

Next, as well as SPRT, applying the weight allocation to the sequential test of WSPRT, the FC takes the following LRT as the decision variable:

$$\begin{array}{c} W_{l}\left(k\right)=\prod_{i=1}^{l}({\frac{P(r_{i}\left(k\right)|H_{1})}{P\left(r_{i}\left(k\right)|H_{0}\right))}}^{w_{i}\left(k\right)} \end{array}$$ (21)

Previous studies have shown that WSPRT functions well in a special scenario where a small fraction of attackers use unsophisticated strategies. Our ultimate objective is to propose a robust CSS against dynamic Byzantine attack, next we will make a comprehensive analysis on deficiencies of WSPRT.

#### Drawbacks of WSPRT

As discussed earlier, the FC has the possibility of being compromised once the condition of (12) is satisfied and no information regarding the primary signal is conveyed in the sequel. Subsequently, the FC’s global decision no longer takes effect as an evaluation criterion for updating TrV.

Another important issue for WSPRT is to assume a somehow simplified attack strategy which can be easily identified. But if in the context of our proposed generic Byzantine attack model, WSPRT has no way of dealing with the negative effect on the network in a long-term run if the attacker selects a proper *α* or *β* to guarantee that TrV is more than −*g*.

In the weight allocation mechanism, when the *i*-th SU’s TrV is lower than −*g* at the *k*-th sensing interval, accordingly, the sensing report is regarded as useless information and is immediately eliminated, i.e., *w*_*i*_(*k*) = 0. In practice, a proper *g* is not easy to select. This radical weight allocation mechanism shows less consideration for shadowing characteristic and multi-path effects during the local spectrum sensing. That is to say, the SU is likely to unintentionally report an incorrect decision due to bad reception conditions, resulting in being misidentified as an attacker.

The above-mentioned shortcomings prevail not only in WSPRT but also in many secure CSS schemes. By this motivation, we present a robust SCSS against dynamic Byzantine attack in the following section.

## Sequential cooperative spectrum sensing

On the basis of above analyses, in this section, we propose a robust SCSS scheme against dynamic Byzantine attack to overcome drawbacks of aforementioned work, which consists of delivery-based assessment, reusing weight allocation and TrV ranking-based sequential test. Proposed SCSS works on a well-defined strategy that will annihilate the impact of Byzantine attack.

### Delivery-based assessment

In CSS, the FC’s global decision plays a key role in assessing the reliability of local reports. Detailedly, the FC compares every local report with the global decision and counts the number of correct reports from each SU. After a few iterations, the FC finds and assigns a TrV to each SU, which is proportional to the correct reports over an observation period [33]. Almost all of TrV-based CSS schemes check the consistency of local reports through the FC’s global decision, including WSPRT mentioned above, but are carried out under a strong assumption that the FC is not compromised. This approach lacks more in-depth consideration on the possibility that the FC might be blind owing to the presence of Byzantine attack, especially the attackers represent the majority, which has already been analyzed in section III, so that the global decision is not reliable as an evaluation criterion.

**Fig 4:**
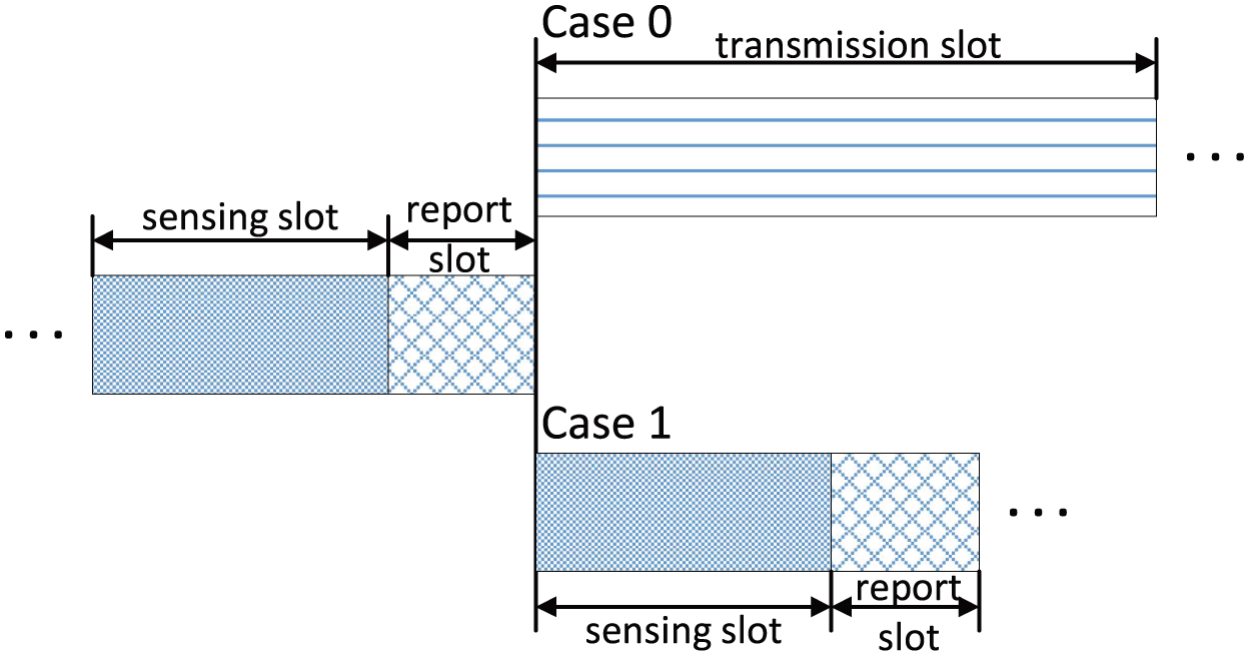
Frame structure with periodic spectrum sensing.

Instead, in this paper, we propose a more simple and effective approach to assess local reports, called delivery-based assessment, which takes advantage of the delivery of the transmitted data of the scheduled SU, but without any alteration to the network. For this aim, we firstly illustrate a frame structure in CRNs for periodic spectrum sensing in Fig 4, where each frame consists of one sensing slot, one report slot, and one transmission slot. Each SU individually implements the local spectrum sensing in the sensing slot, and submits individual sensing result to the FC in the report slot, thus the FC is responsible for making a global decision on the presence or absence of the primary signal based on its received information and determines whether SUs can access idle channels or not. We extend the delivery-based assessment approach to two cases of the global decision as follows.

#### Case 0

When the global decision declares the licensed channel as idle, which implies that one of SUs can be scheduled to access the unused channel for data transmission. The successful delivery of the transmitted data reveals that the global decision was correct and that the channel is actually unused. If the transmitted data cannot be successfully delivered (because of the harmful interference between the primary and secondary network), the global decision is identified as incorrect, and the channel is actually occupied [34].

#### Case 1

When the global decision declares the licensed channel as busy, all SUs need to switch to another channel and sense its availability in the next sensing interval. If there is no data transmission which represents the global decision was correct, otherwise definitely incorrect. The reason is that the reliable SUs have switched another channel to sense, the unused channel must be selfishly occupied by attackers if there exists the successful transmitted data.

Doubtlessly, in both cases, the FC has realized the actual channel status, which can be used to assess all the received local decisions as correct or not. As an evaluation criterion, data delivery is much more reliable than the global decision, even in the worst scenario where Byzantine attack makes the FC blind.

In terms of implementation, the delivery-based assessment approach can be easily applied in a centralized CRN, where SUs individually access the spectrum, the data delivery can be verified by an additional monitoring process during data transmission performed by the FC itself. Notice that the monitoring process is much easier than spectrum sensing since the transmitting user is known at the FC [34]. Hence, according to the delivery of the transmitted data of the scheduled SU, the TrV renewal of (19) can be rewritten as

$$\begin{array}{c} T_{i}\left(k\right)=T_{i}\left(k-1\right)+{\left(-1\right)}^{\left(r_{i}\left(k\right)+\hat{d}\left(k\right)\right)} \end{array}$$ (22)

where $\hat{d}\left(k\right)$ represents the channel status information of delivery-based assessment.

### Reusing weight allocation

Via delivery-based assessment, attack identification policy is easily carried out by TrV-based algorithm where, on the one hand, the local reports of SUs with large TrVs are assigned with large weighting coefficients, while, on the other hand, SUs with TrVs lower a pre-determined value are removed from the fusion process at the FC. Such a method has been used by the majority of research, and can minimize the impact of attack on the network to the maximum extent, while this conservative defensive strategy is likely to misidentify the reliable SU as the attacker because the reliable SU may experience fading or happen to be shadowed. Further, a large number of attackers exist in the network, the advantages of CSS will greatly decline if they are also removed in accordance with attack identification policy.

This observation motivates us to come up with an alternative way to deal with malicious reports rather than jettison. Observing that attack identification policy is based on such a premise that the attacker’s report is useless sensing information. In our work, reusing malicious report is chosen as an objective for turning waste into treasure which represents the first effort in this direction to the best of our knowledge. The main benefit of reusing malicious report as an objective is to enhance the CSS performance since the attacker may convey some useful information about the PU status. Based on this consideration, we now propose a novel reusing allocation scheme for the weight of the local sensing report.

We define a pairwise of correct decision ability, the presence and absence decision ability, which respectively denote the ability of correctly deciding the PU’s presence and absence. The presence and absence decision ability of the *i*-th SU can be respectively described as

$$\begin{array}{c} A_{i}^{1}\left(k\right)=\frac{k_{i}^{1}}{k^{1}},A_{i}^{0}\left(k\right)=\frac{k_{i}^{0}}{k^{0}} \end{array}$$ (23)

where *k*^1^ and *k*^0^ represent the cumulative round of the presence and absence of the PU after the *k*-th sensing interval, $k_{i}^{1}$ and $k_{i}^{0}$ denote the cumulative round of correctly deciding the presence and absence of the PU at the *i*-th SU, which can be counted by delivery-based assessment. For simplicity of denotation, $A_{i}^{1}\left(k\right)$ and $A_{i}^{0}\left(k\right)$ are generally referred to as $A_{i}^{x}\left(k\right)$, while $\frac{k_{i}^{1}}{k^{1}}$ and $\frac{k_{i}^{0}}{k^{0}}$ are referred to as $\frac{k_{i}^{x}}{k^{x}}$, where *x* is either 1 or 0.

The starting point of reusing weight allocation in that when $A_{i}^{x}\left(k\right)$ is in close proximity to 0, the *i*-th SU can be regarded as an attacker launching “always attack”. For examples, when $A_{i}^{1}\left(k\right)$ is near to 0, the *i*-th SU is definitely an AN attacker, and when $A_{i}^{0}\left(k\right)$ moves towards 0, it is an AY attacker. Therefore, at the end of the *k*-th sensing interval, the *i*-th SU with the correct decision ability higher than 0.85 remains and will be allocated with the weight $\frac{k_{i}^{x}}{k^{x}}$ to prepare for the next sensing interval. If the correct decision ability is inferior to 0.15, the *i*-th SU’s report in the next sensing interval will be reserved with the weight $1-\frac{k_{i}^{x}}{k^{x}}$. Such an approach innovatively turns attackers’ sensing reports into useful sensing information to enhance the cooperative performance. Otherwise, for those SUs with the correct decision ability between 0.15 and 0.85, the weights of their reports are set to be 0, because they are interferential information for the FC. Thus, the reusing weight allocation is given by

$$\begin{array}{c} w_{i}^{x}\left(k\right)=\left\{ \begin{array}{c} 1-\frac{k_{i}^{x}}{k^{x}},A_{i}\left(k\right)\leq 0.15\\ \frac{k_{i}^{x}}{k^{x}},A_{i}^{x}\left(k\right)\geq 0.85\\ 0,\text{otherwise} \end{array}\right. \end{array}$$ (24)

In (24), two factors account for selecting the range of $w_{i}^{x}\left(k\right)=0$. On one side, the larger the range is set, the more the useful sensing information from attackers will be excluded. On the other side, the smaller range eventually leads to the more opportunities for attackers to undermine CSS..

It is worth noting that SUs being identified attackers are not simply eliminated in our reusing weight allocation, but the weights of their reports are set to 0. Even if the reliable SU may be temporarily mistaken for an attacker due to the unfavorable channel condition, there is no possibility of being eliminated. For those attackers, though we do not deal with them as strictly as other algorithms, the restrictions on them will be involved in the scheduling policy in the next subsection.

### TrV ranking-based sequential test

It is known that CSS is the large communication resource requirement for reporting sensing results, particularly, in a large CRN [35]. As discussed earlier, though it has shown that SPRT can reduce the requirement of samples, the presence of Byzantine attack may make the FC require a large sample size to make a global decision. For this reason, on the premise of ensuring the network robustness, we provide a TrV ranking-based sequential test in order to reduce the overhead, the total processing time and the energy consumption for CSS.

Since SPRT does not strictly specify the order in which the FC fuses the reports from SUs, WSPRT is prone to become stuck and even deadlock triggered by attackers. Therefore, we rank SUs’ TrVs in a descending order, and the SU with a higher TrV is prior to computes the likelihood ratio and compares it with the lower threshold *λ*_*l*_ and upper threshold *λ*_*u*_, until the sequential test terminates. After the FC outputs a global decision, reports of the remaining SUs are no longer submitted to the FC. In such a way, our TrV ranking-based sequential test only requires less report samples than WSPRT to make a more reliable decision, while shortening reporting time and prolonging data transmission time in a fixed frame slot. Consequently, it can significantly improve the efficiency of spectrum sensing and secondary network throughput.

Based on the key components, our proposed SCSS scheme at the *k*-th sensing interval can be described in the following SCSS.

**Algorithm 1** SCSS

1: **Initial** *T*_*i*_(1) = 0, $A_{i}^{x}\left(1\right)=1$, i=1,2,3,…N.

2: **for** *k* to sensing limit **do**

3:  Rank TrVs *T*_1_(*k*), *T*_2_(*k*), …, *T*_*N*_(*k*) of *N* SUs in a descending order.

4:  Rank the local reports *r*_1_(*k*), *r*_2_(*k*), …, *r*_*N*_(*k*) of *N* SUs in a descending order of TrV.

5:  *W*_*l*_(*k*) = 1.

6:  **for** *i*: *N* **do**

7:   Allocate the *i*-th SU’s weight $w_{i}^{x}\left(k\right)$ according to its correct decision ability $A_{i}^{x}\left(k\right)$.

8:   **if** *r*_*i*_(*k*) = 1 **then**

9:    **if** $A_{i}^{1}\left(k\right)\leq 0.15$ **then**

10:     $w_{i}^{1}\left(k\right)=1-\frac{k_{i}^{1}}{k^{x}}.$

11:    **end if**

12:    **if** $A_{i}^{1}\left(k\right)\geq 0.85$ **then**

13:     $w_{i}^{1}\left(k\right)=\frac{k_{i}^{1}}{k^{x}}.$

14:    **end if**

15:    **if** $0.15<A_{i}^{1}\left(k\right)<0.85$ **then**

16:     $w_{i}^{1}\left(k\right)=0.$

17:    **end if**

18:    $W_{l}\left(k\right)=\prod_{i=1}^{l}{\left(\frac{P(r_{i}\left(k\right)|H_{1})}{P(r_{i}\left(k\right)|H_{0})}\right)}^{w_{i}^{1}\left(k\right)}$.

19:   **else**

20:    **if** $A_{i}^{0}\left(k\right)\leq 0.15$ **then**

21:     $w_{i}^{0}\left(k\right)=1-\frac{k_{i}^{0}}{k^{x}}.$

22:    **end if**

23:    **if** $A_{i}^{0}\left(k\right)\geq 0.85$ **then**

24:     $w_{i}^{0}\left(k\right)=\frac{k_{i}^{0}}{k^{x}}.$

25:    **end if**

26:    **if** $0.15<A_{i}^{0}\left(k\right)<0.85$ **then**

27:     $w_{i}^{0}\left(k\right)=0.$

28:    **end if**

29:    $W_{l}\left(k\right)=\prod_{i=1}^{l}{\left(\frac{P(r_{i}\left(k\right)|H_{1})}{P(r_{i}\left(k\right)|H_{0})}\right)}^{w_{i}^{0}\left(k\right)}$.

30:   **end if**

31:   **if** *λ*_*l*_ < *W*_*s*_(*k*) < *λ*_*u*_ **then**

32:    go to step 6.

33:   **end if**

34:   **if** *W*_*l*_(*k*) ≥ *λ*_*u*_ **then**

35:    accept *H*_1_, then go to step 41.

36:   **end if**

37:   **if** *W*_*l*_(*k*) ≤ *λ*_*l*_ **then**

38:    accept *H*_0_, then go to step 41.

39:   **end if**

40:  **end for**

41:  $T_{i}\left(k\right)=T_{i}\left(k-1\right)+{\left(-1\right)}^{\left(r_{i}\left(k\right)+\hat{d}\left(k\right)\right)}$.

42:  Update $A_{i}^{x}\left(k\right)$ according to delivery-based assessment.

43:  The high TrV SUs are given priority to allocate spectrum resources.

44: **end for**

After SCSS, the next question that arises is how to dispose of attackers. Attacker identification can be done through delivery-based assessment, even we can accurately differentiate whether an attacker launches FAA or MDA according to a pairwise of correct decision ability. But attacker identification is not of our original intention. As stated in our reusing weight allocation, none of attackers are excluded from the network. But this does not mean that they can attain the goal of malicious interfering with the primary network and occupying spectrum resources. Given that it is unfair to provide the same opportunity for all SUs to access the spectrum, we provide a simple and effective scheduling policy to realize a fair spectrum resource allocation principle. The scheduling policy distributes scheduling probability to each SU according to its TrV, the SU with a higher TrV has a high scheduling probability for data transmission. Such a fair scheduling policy acts as a punishment for attackers and a reward for reliable SUs [34], which not only enhances the collaboration performance by reuse of Byzantine data but also ensures the network security under the constraint of malicious behaviors.

## Simulation results

In this section, we perform a few experiments based on simulated data to validate our Byzantine attack analysis and demonstrate the performance of the proposed SCSS by comparison with other data fusion technologies.

In voting rule, three variants of Or, And, Majority rule are simulated. In Bayesian detection, *λ*_*Bayes*_ is calculated by the prior probabilities of hypotheses, i.e., *P*(*H*_0_) = 0.7 and *P*(*H*_1_) = 0.3. The costs are assigned as: *C*_00_ = *C*_11_ = 0, *C*_10_ = 1 and *C*_01_ = 10. In N-P test, *λ*_*NP*_ is set to be 100 in comparison with a small *λ*_*Bayes*_. Another simulated fusion techniques are SPRT, WSPRT, and SCSS. The parameters in these three fusion techniques used in the simulation are *A* = 10^−3^ and *B* = 10^−4^. The simulations are based on the Monte-Carlo method with 10000 iterations.

In order to evaluate the detection performance, we utilize the global error probability *Q*_*e*_ which is defined as follows:

$$\begin{array}{c} Q_{e}=P\left(H_{0}\right)Q_{f}+P\left(H_{1}\right)Q_{m} \end{array}$$ (25)

where *Q*_*f*_ is the global false alarm probability and *Q*_*m*_ is the global miss detection probability. Other than the error probability, we also are interested in an additional performance metric, which is the average number of samples that SUs submit to the FC that used for making a global decision over an observation period, and it measures the overhead of a particular data fusion technique [8]. For voting rule, Bayesian detection and N-P test, a fixed sample size is required for making a global decision, while the number of samples of SPRT-based CSS scheme changes. Therefore, we only focus this metric for SPRT, WSPRT and SCSS.

### Simulation environment

The $\bar{PL}\left(d\right)$ in (1) employs the Hata model, which has been different application environments. Given that CRNs are more likely to be actually applied in rural areas because the licensed spectrum is not fully utilized. The standard formula for empirical path loss in open rural areas under the Hata model is given by [23] [36]

$$\begin{array}{c} {\bar{PL}}_{rural}\left(d\right)={\bar{PL}}_{urban}\left(d\right)-4.78{\left(log_{10}\left(f_{c}\right)\right)}^{2}+18.33log_{10}\left(f_{c}\right)-40.98 \end{array}$$ (26)

$$\begin{array}{ccc} {\bar{PL}}_{urban}\left(d\right) & = & 69.55+26.16log_{10}\left(f_{c}\right)-13.82log_{10}\left(h_{t}\right)-a\left(h_{r}\right)\\ & & +\left(44.9-6.55log_{10}\left(h_{t}\right)\right)+18.33log_{10}\left(f_{c}\right)-40.98 \end{array}$$ (27)

$$\begin{array}{c} a\left(h_{r}\right)=\left(1.1log_{10}\left(f_{c}\right)-0.7\right)h_{r}-\left(1.56log_{10}\left(f_{c}\right)-0.8\right) \end{array}$$ (28)

where ${\bar{PL}}_{urban}\left(d\right)$ is the standard formula for empirical path loss in urban areas under the Hata model, *f*_*c*_ is the signal frequency, *h*_*t*_ is the effective transmitter antenna height in meters, *h*_*r*_ is the effective receiver antenna height in meters, and *a*(*h*_*r*_) is the correction factor for effective mobile antenna height which is a function of the size of the coverage area.

In our simulation, the related parameter settings are also used in [6] [7]. Assume that the PU is at frequency of 617MHz, *h*_*t*_ = 50*m*, *h*_*r*_ = 1*m*. At the transmitter/incumbent side, and the effective isotropic radiated power (EIRP) is assumed to be 80kW. Each receiver has a typical sensitivity of −92dBm, which is the minimum power for a signal to be detected. For the noise power, the typical value of *n*_0_ in the considered band frequency −106dBm is used. For the deviation part of the log-normal shadowing path loss model and noise, we adopt *σ* = *σ*_0_ = 11.8. Otherwise, *d*_0_ = 100*m*, *l* = 4.4, *r* = 1000*m*. For the sake of easy presentation, *ψ* is set 0. The distance between SUs is negligible compared to the distance between SUs and the PU.

### Static scenario

Due to that most of CSS methods do not involve mobile scenario, we start with a static scenario in order to build a fair comparison framework, and fix *d* = 2000, *N* = 100 while changing attack types and varying *ρ* from 0 to 0.9. To better reflect the nature of the attacker, an elusive behavior which attackers randomly launch Byzantine attack is simulated over different observation periods.

### Always attack

**Fig 5:**
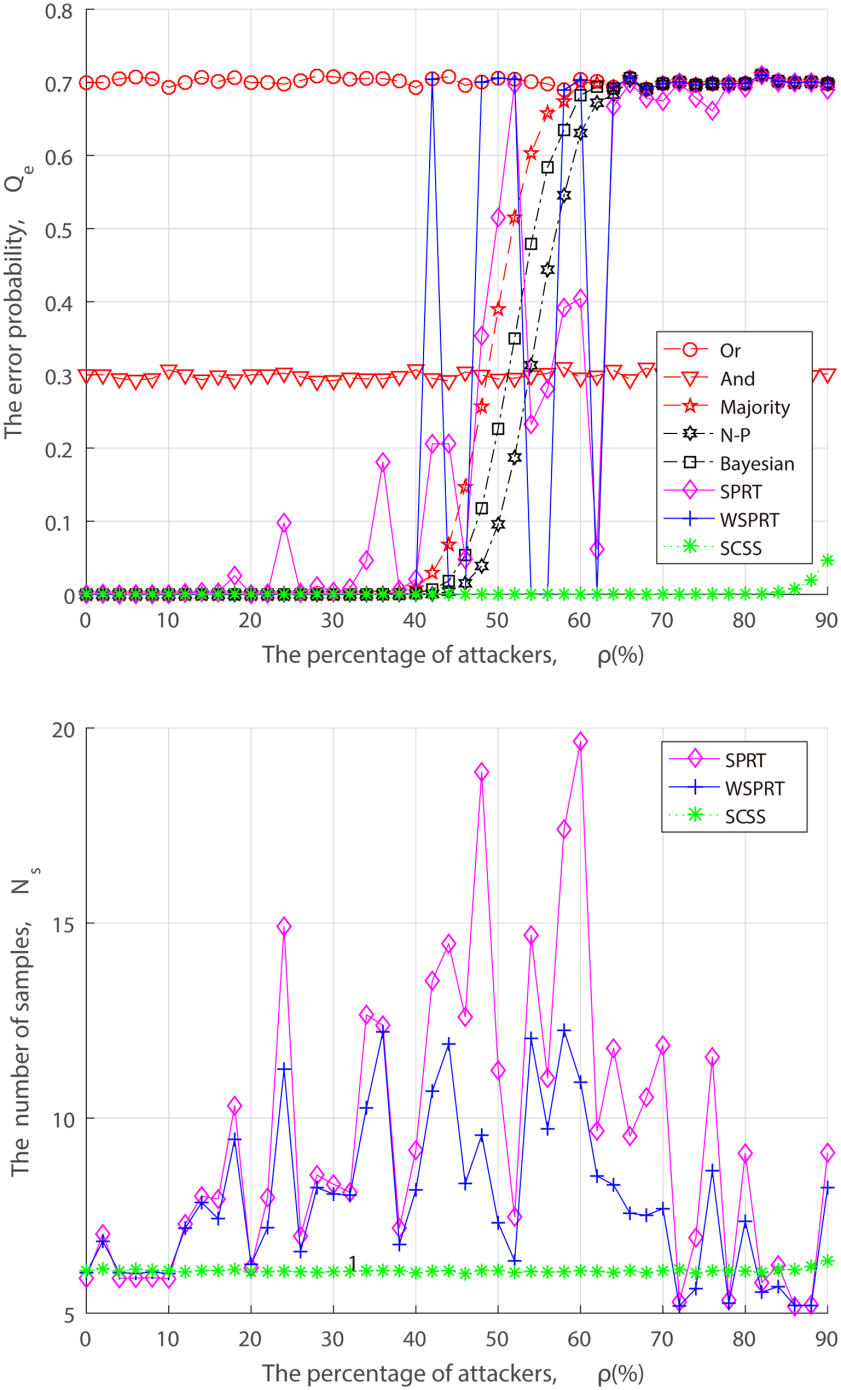
Performance of fusion techniques with varying percentage of AY attackers. (a) the error probability. (b) the number of samples.

**Fig 6:**
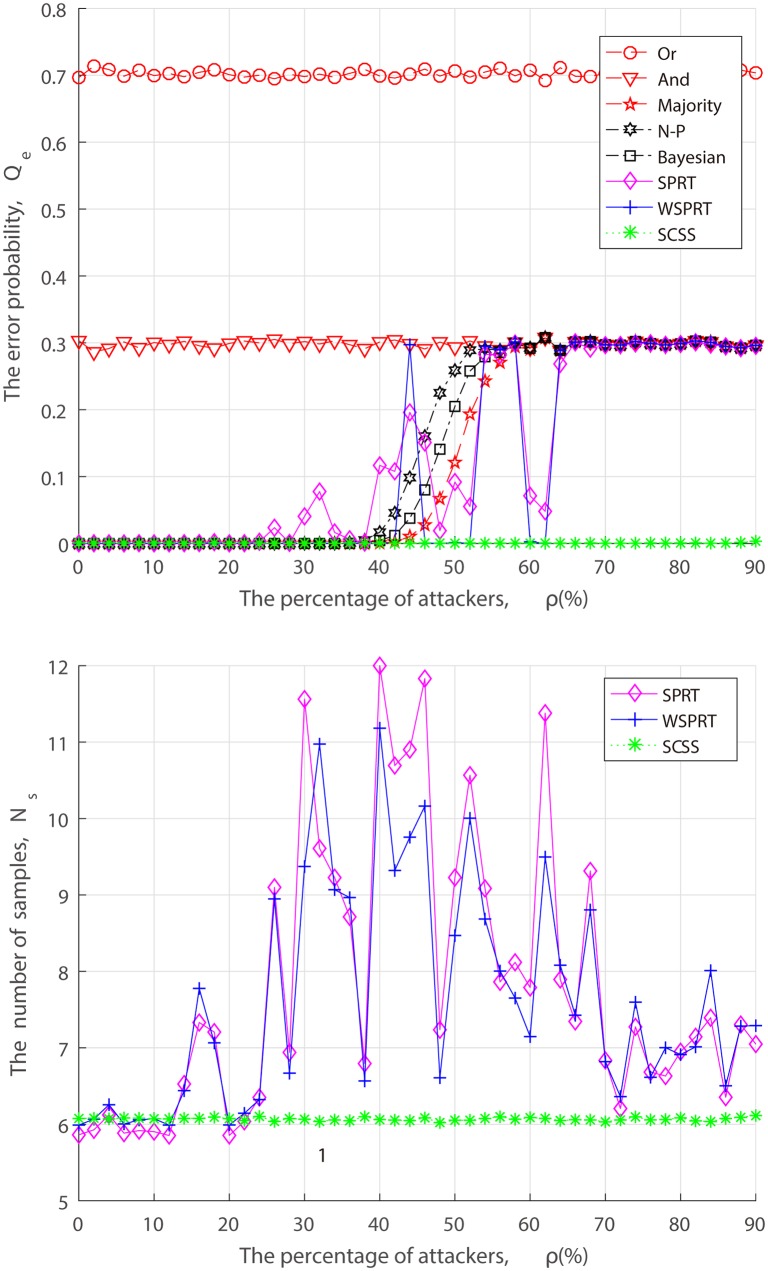
Performance of fusion techniques with varying percentage of AN attackers. (a) the error probability. (b) the number of samples.

**Fig 7:**
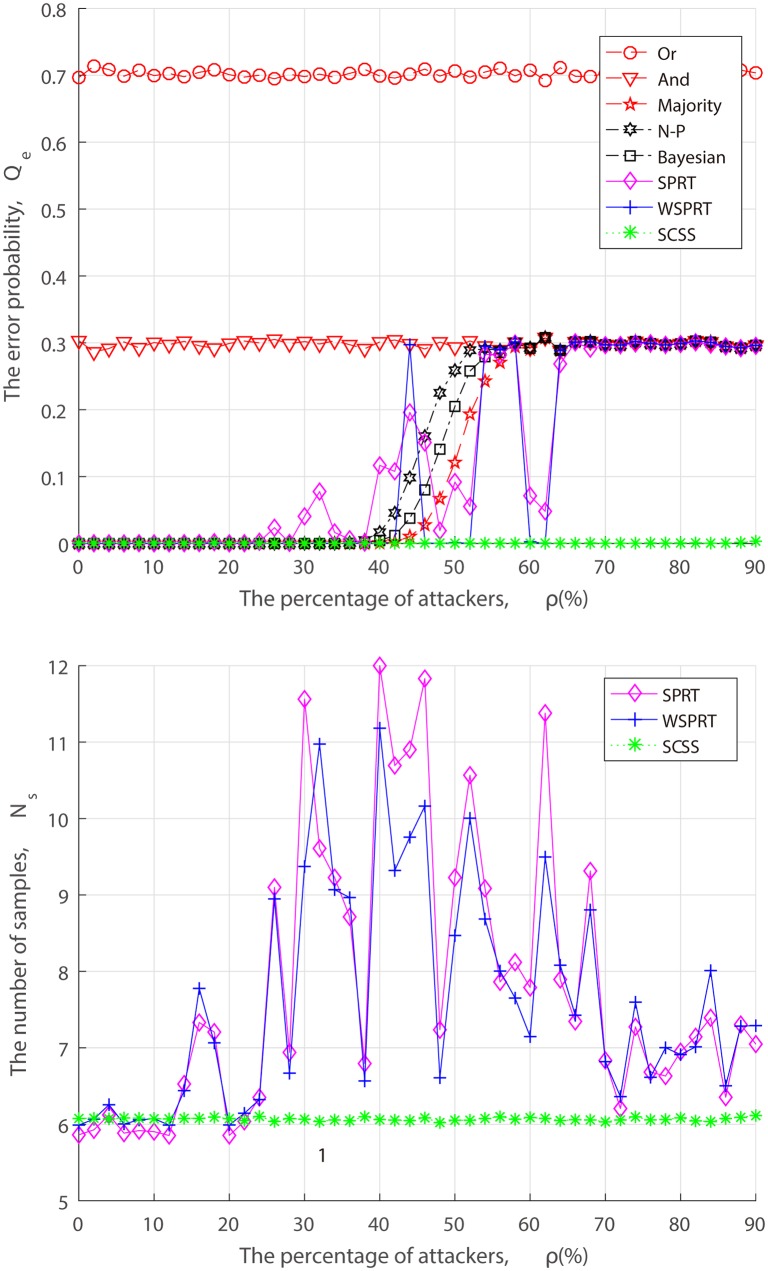
Performance of fusion techniques with varying percentage of AF attackers. (a) the error probability. (b) the number of samples.

Under the attack of AY, AN and AF, the relationship between the performance and the attack ratio is illustrated in Figs 5–7 for different fusion techniques. It is evident that the error probability of Or rule and And rule respectively stay at 0.7 and 0.3 from the beginning, since they approximately always declare the channel as either busy or idle. Other fusion technologies can guarantee the detection accuracy in the presence of a few attackers, while the error probabilities have risen to varying degree as attackers increase.

In a series of “always attack” simulations, the error probability of SPRT and WSPRT fluctuate in varying degrees, as depicted in Figs 5a–7a, the main difference lies in the fluctuation range and fluctuation magnitude. The larger fluctuation range of SPRT is not a surprise, and is in fact a direct consequence of its lack of consideration of attack, as it does not differ in receiving any reports, including malicious ones, which amplifies the negative effect of attack. Unlike SPRT, the weight allocation of WSPRT can suppress malicious behaviors, but only when there exist a few attackers. Our comparative results clearly reveal that WSPRT is extremely sensitive to the number of attackers, and the higher the attack ratio, the higher the possibility of the FC being blinded, resulting in the failure of its weight allocation. For Majority, Bayesian detection and N-P test, their error probabilities are not as volatile as SPRT and WSPRT but rapidly increases once the attack ratio reaches the critical value of 50%. By comparison, it can be found that SCSS is not affected by blind condition. This is attributed to the fact that delivery-based assessment to assess local reports is more reliable than the FC’s global decision.

As for the sample size, Figs 5b–7b show that there is no significant difference between three SPRT schemes at the outset. But with the increasing attackers, the sample size of SPRT and WSPRT become larger, which is followed by the unstable detection performance. Obviously, the negative impact of malicious reports is gradually expanding. As the proportion of attackers further increases, the number of samples required for SPRT and WSPRT gradually decreases and the error probability increases, which indicates that the FC begins to be compromised at this time. In contrast, the sample size of SCSS is basically maintained at 6 for various proportion of attackers.

Ultimately, as can be seen from Figs 5a–7a, the error probability of Majority, N-P, Bayesian, SPRT, WSPRT for AY attack converges to 0.7, the error probability for AN attack and AY attack converge to 0.3 and 1, respectively. The reason for this phenomenon is that the false alarm probability and miss detection probability of an AY attacker are 1 and 0, respectively, while an AN attacker are the opposite and an AF attacker are both 1. According to (25), the convergence performance that is consistent with simulation results can be easily obtained. As anticipated, the performance of SCSS bests that of other fusion techniques regardless of what proportion of “always attack”. Even though the percentage of attackers is more approachable to 85%, the sample size and the error probability for SCSS have both risen, but they are still impressive. There are two reasons for this. First, delivery-based assessment lays a foundation for accuracy detection of SCSS. Second, the principle of TrV ranking-based sequential test ensures stable detection performance in an arbitrary attack ratio.

### Dynamic attack

From the perspective of attackers, when launching the attack, they have to take into the factors of attack cost and attack risk account, and may act in a strategic manner to dynamically adjust the FAA and MDA probability rather than the simplified “always attack”. Thus, we simulate the error probability and sample size of different fusion techniques under dynamic attack strategy in the context of three combinations of FAA probability *α* and MDA probability *β*.

**Fig 8:**
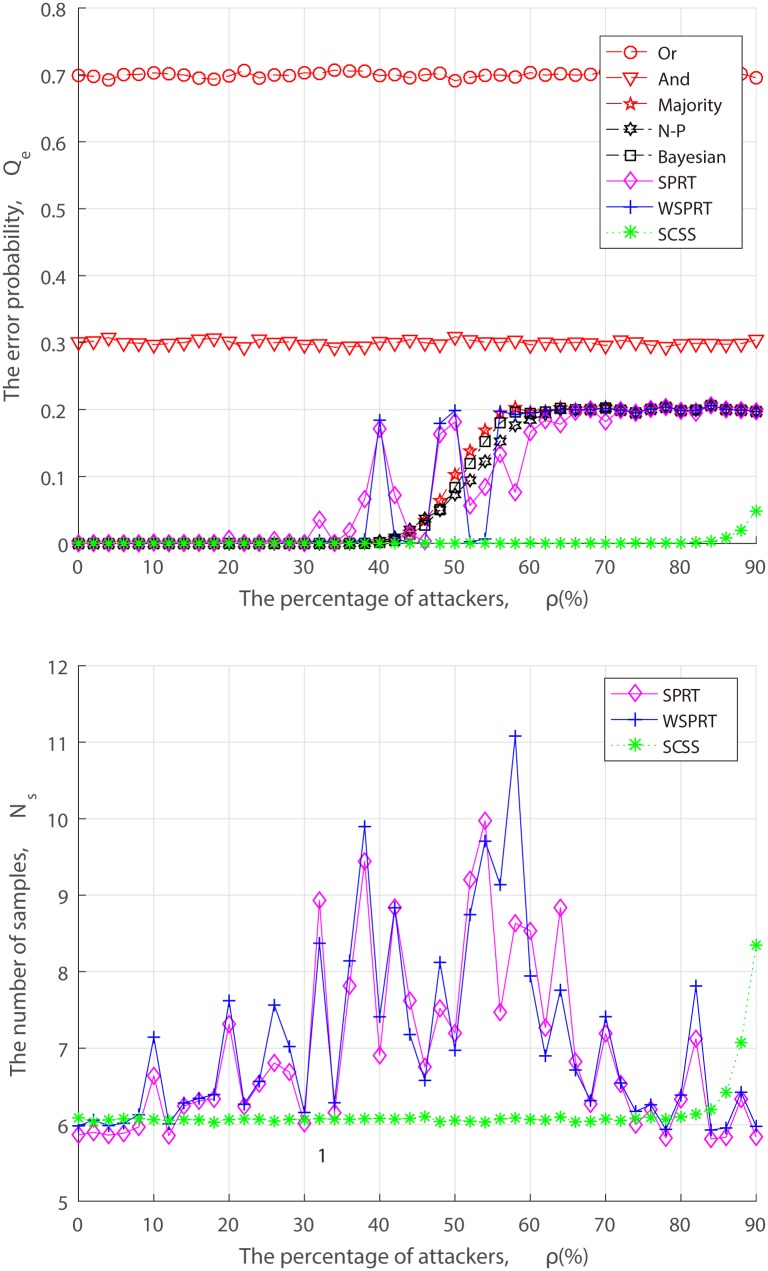
Performance of fusion techniques under (*α*, *β*) = (0.2, 0.2). (a) the error probability. (b) the number of samples.

**Fig 9:**
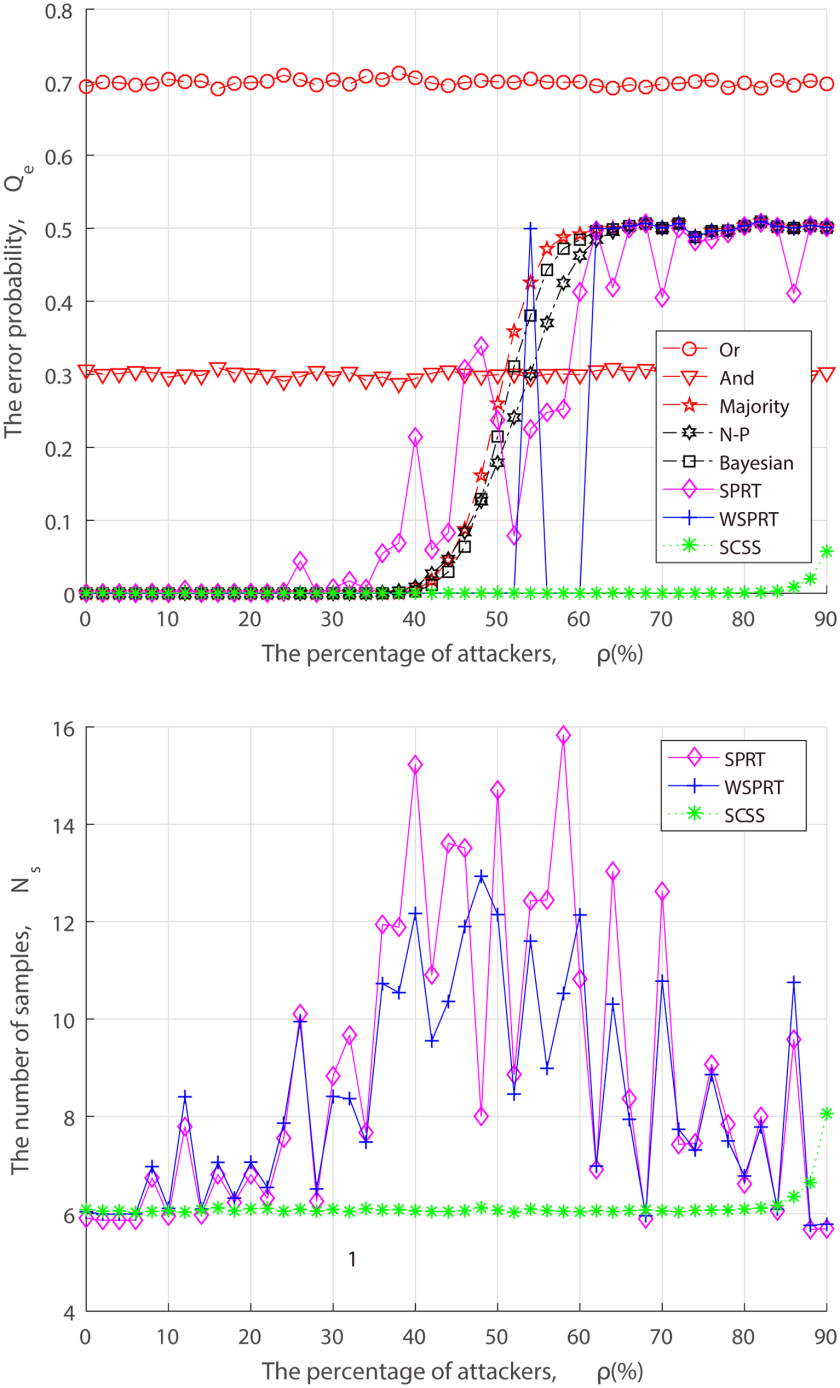
Performance of fusion techniques under (*α*, *β*) = (0.5, 0.5). (a) the error probability. (b) the number of samples.

**Fig 10:**
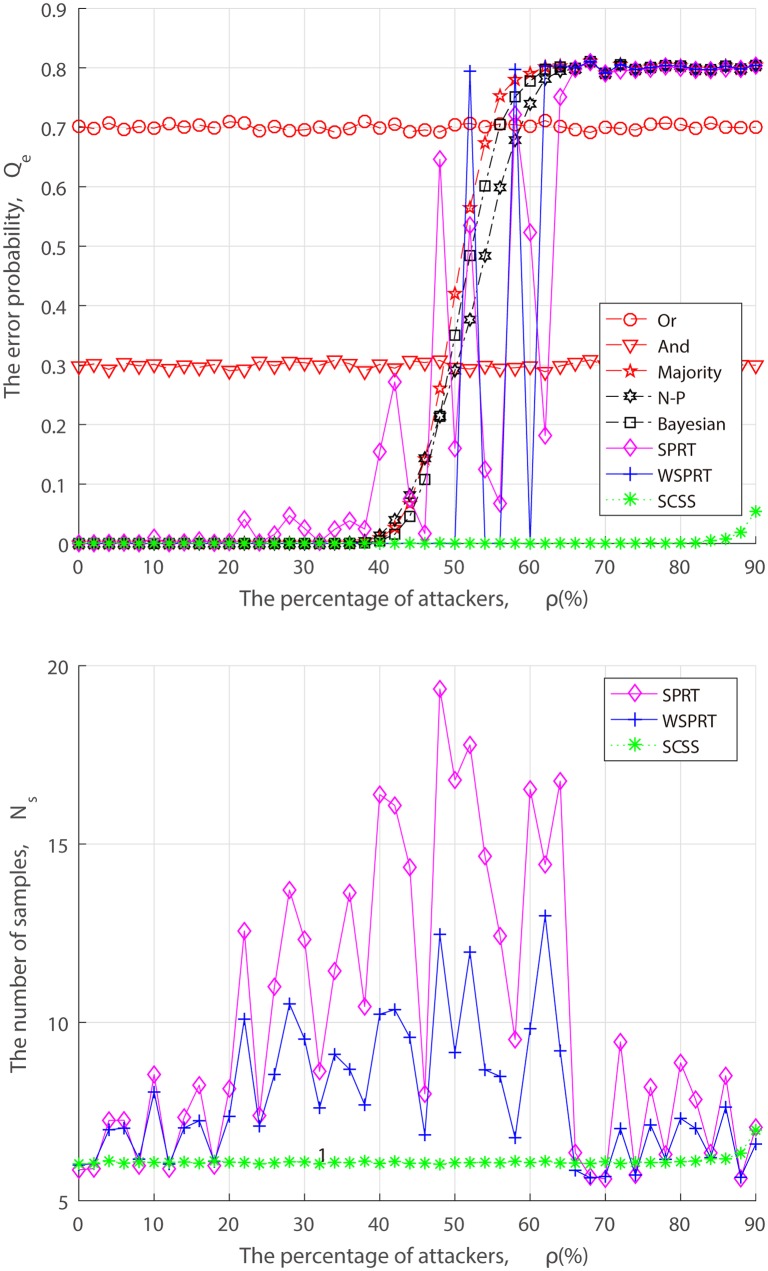
Performance of fusion techniques under (*α*, *β*) = (0.8, 0.8). (a) the error probability. (b) the number of samples.

In the set of simulations shown in Figs 8–10, when attackers launch FAA and MDA in (*α*, *β*) = (0.2, 0.2), (0.5, 0.5), (0.8, 0.8). Performance changes of different fusion technologies are similar to that of “always attacks”. As we can see from Figs 7a–10a, when attack strength is low (attack probability is small), although attackers cannot make the FC blind, as the fraction of attackers increases, the error probability finally converges to the attack probability. With the exception of Or rule, And rule and SCSS, the error probability of others converge to *P*(*H*_0_)*α* + *P*(*H*_1_)*β*, such as, when (*α*, *β*) = (0.2, 0.2), the error probability converges to *P*(*H*_0_) ∗ 0.2 + *P*(*H*_1_) ∗ 0.2 = 0.2. In spite of some attack strategies, the FC is not completely blind, but the system is always in a state of low performance, which inevitably causes long-term interference to the primary network and selfishly occupy the idle channel by attackers. Especially, when AF attacker is introduced, not surprisingly, 50% attackers can make the FC blind as illustrated in Fig 7a. This result confirms our theoretical analysis of (12). Nevertheless, no matter what an attack strategy (attack ratio, attack probability) the attacker takes, the detection performance of our proposed SCSS exceeds other data fusion technologies, and is still impressive even in the case of a high malicious presence, adding only a few samples.

From Figs 5 to 10, it is concluded that the unstable performance of SPRT and WSPRT are caused by the elusive attack behavior. In order to ensure reliability of simulation results, we conduct 100 simulation experiments to take the average results from S1 Fig to S6 Fig. The only difference from our previous simulation results is that the performance of SPRT and WSPRT has become relatively smooth. The common thing is that their better performance is comparable to that of SCSS in the presence of a few attackers. However, as attackers increase, SCSS can still maintain good detection performance, but only increases the number of samples, while the detection performance by SPRT and WSPRT dramatically decreases with the drop in samples. This again confirms the validity of our proposed SCSS.

So far, we have presented the performance of different data fusion technologies under several combinations of FAA probability and MDA probability. Yet another state of data fusion to be considered is SU density. For a deeper insight into how the proposed scheme benefits from malicious reports to achieve better performance, we observe the impact of SU density on the performance in terms of various FAAs and MDAs. Since the results and analyses show that existing data technologies expose a high vulnerability for a high attack ratio. Hence, we only focus on SCSS in the following simulations.

### Impact of SU density

The value of *N* in fact decides the SU density in CRNs. In order to observe the impact of the SU density on the error probability and the number of samples, we fix *d* = 1500 in the presence of 80% attackers while varying *N* from 30 to 120 at an interval of 10.

**Fig 11:**
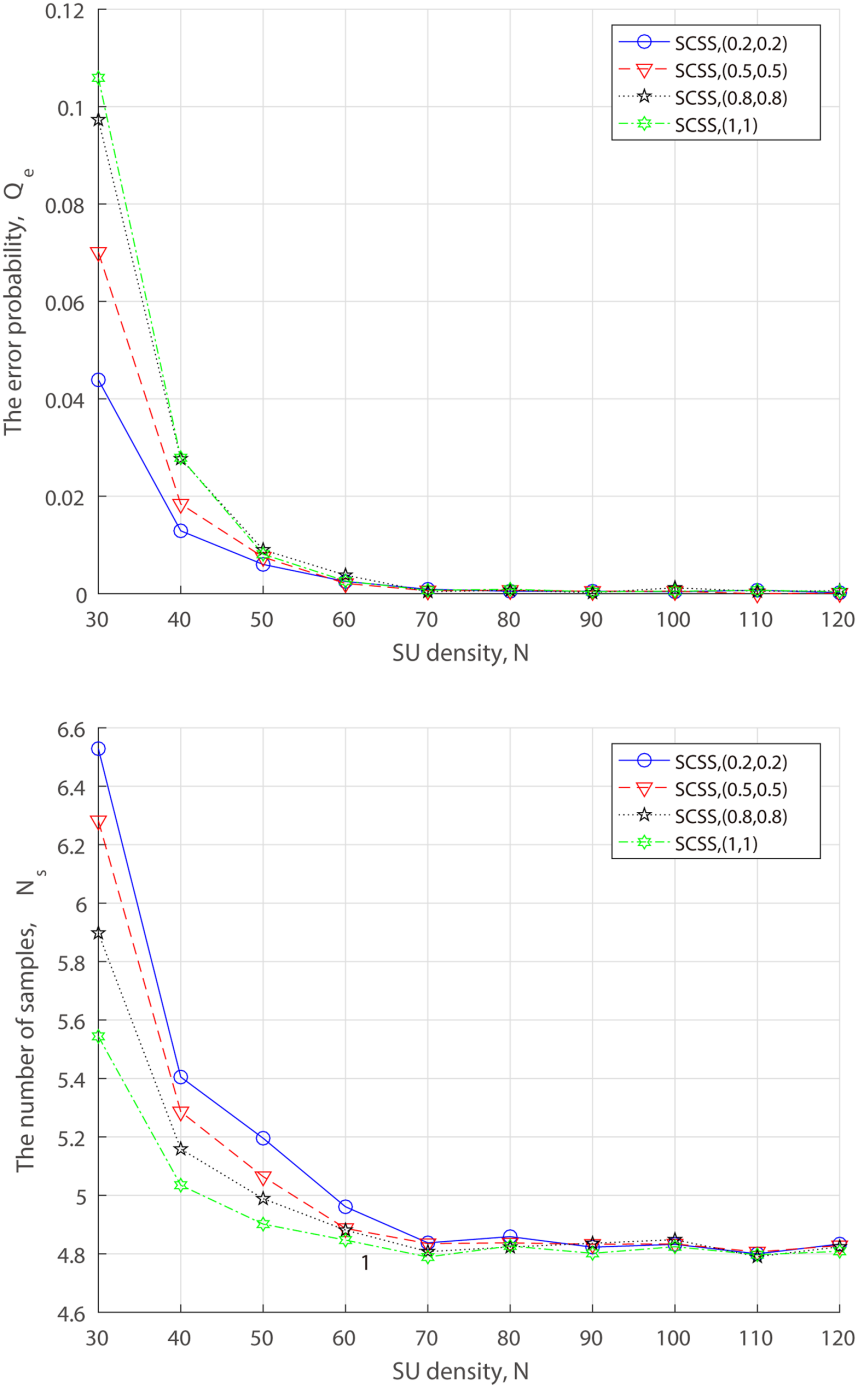
Performance of SCSS v.s. SU density for varing attack probability. (a) the error probability. (b) the number of samples.

Fig 11 shows that whatever the probability of FAA and MDA in the network is, the higher SU density always results in that fewer samples generate a more accurate decision. Furthermore, one interesting effect we can also see is that the higher attack strength makes SCSS only require less samples at a fixed *N* while ending up with a similar detection accuracy with the lower attack strength. To be specific, if there exist enough SUs to cooperate, the impact of attack strength on the error probability can be neglectable. There is no doubt that this is due to the success of our proposed reusing weight allocation scheme using malicious reports, and the higher the attack strength, the more obvious the attack features, the more valuable malicious reports. As a consequence, SCSS needs the least samples for AF attack, while the most samples for (*α*, *β*) = (0.2, 0.2).

### Mobile scenario

Mobility is one of the most important factors in wireless systems because it affects numerous network characteristics. In the real implementation of CRNs, the mobility may be of our concern. To this end, we assume the initial waypoint *d*_1_ = 1500*m* and *N* = 100, SU velocity varies from 10 to 40m/s within the range of the network area and a maximum idle time of 120s.

**Fig 12:**
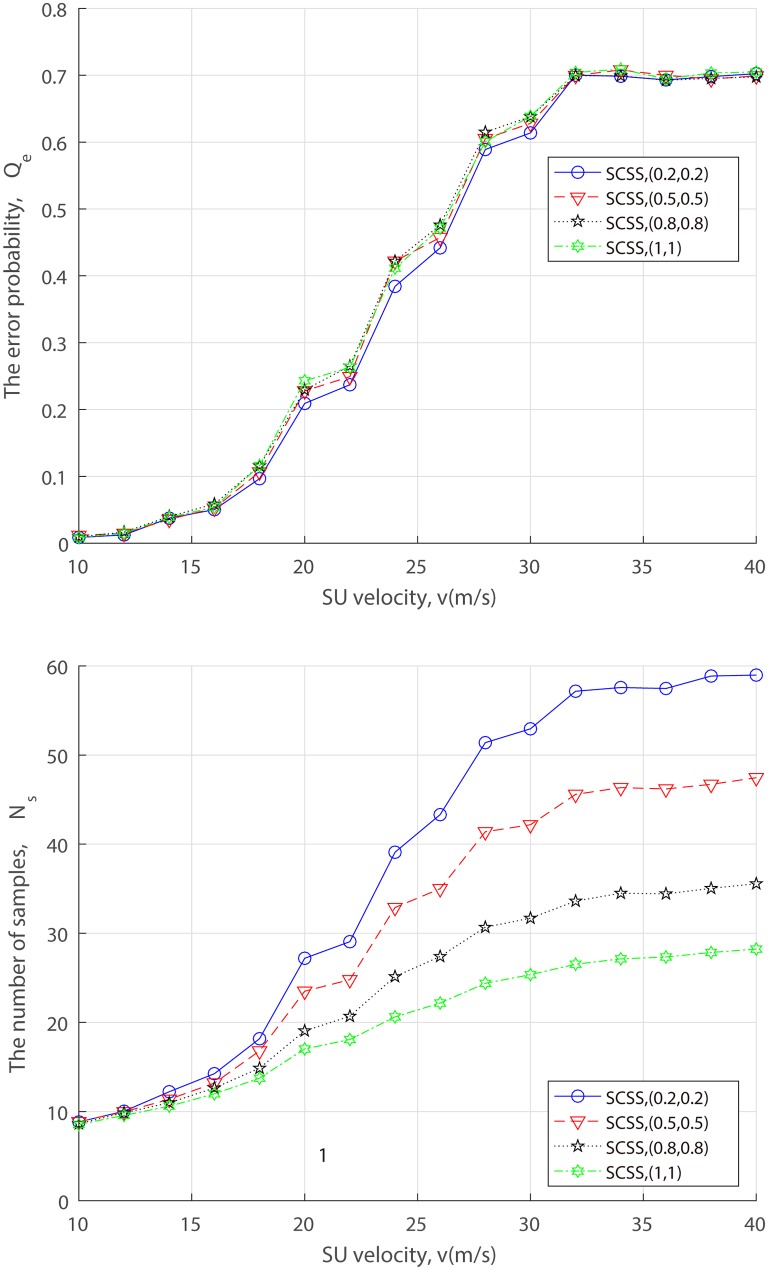
Performance of SCSS v.s. SU velocity for varing attack probability. (a) the error probability. (b) the number of samples.

In Fig 12, we simulate the error probability and sample size of SCSS in terms of varying attack probability when there exist 80% attackers. It can be observed from Fig 12b that the faster the SU velocity, the greater the number of samples. This is reasonable, because the SU velocity deteriorates the local sensing performance and SCSS has to require more decision samples in support of the FC’s decision. Whereas different from the negative effect of SU velocity, Fig 12a shows that attack strength has little effect on the error probability for a fixed SU velocity, which corroborates the correctness and effectiveness of reusing malicious reports once again.

## Conclusions

In this paper, we have proposed a sequential test against dynamic Byzantine attack for mobile networks. Unlike the previous static scenario and simplified attack strategies, we consider spectrum sensing problem in a more realistic mobile scene and propose a generic attack model (FAA and MDA) by analyzing malicious behaviors, aiming at evolving dynamic attack strategies from the attacker’s perspective. The blind problem is the most representative Byzantine sophistication in dynamic attack strategy, and a closed form expression of the blind condition is also derived. Further, we give a brief of introduction to existing data fusion techniques including voting rule, hypothesis test and decompose drawbacks of WSPRT. To deal with these issues, our proposed SCSS, which takes advantage of delivery-based assessment to assess local reports as well as reusing weight allocation to benefit from malicious reports, operates sequential test in a TrV ranking order. Furthermore, a fair scheduling policy is added as spectrum resource allocation principle to enhance the collaborative performance and secure the network. Finally, simulation results show that, compared to existing data fusion technologies, the superiority of SCSS with respect to the error probability is evident at the expense of a small sample size, which proves the high performance of the proposed policy under dynamic attack, even in the case of Byzantine attack satisfying the blind condition. Simultaneously, the density and mobility of SU show the positive and negative effects on SCSS, respectively.

There are still many interesting questions that remain to be explored in the future work, such as the fast accuracy malicious detection in a high-speed mobile scenario and the optimal attack strategy investigation for smart Byzantines.

## Supporting information

S1 Fig The average results for Fig 5. (ZIP)

S2 Fig The average results for Fig 6. (ZIP)

S3 Fig The average results for Fig 7. (ZIP)

S4 Fig The average results for Fig 8. (ZIP)

S5 Fig The average results for Fig 9. (ZIP)

S6 Fig The average results for Fig 10. (ZIP)

## References

[1] Akbari M, Ghanbarisabagh M. A novel evolutionary-based cooperative spectrum sensing mechanism for cognitive radio networks. Wireless Personal Communications. 2014;79(2):1017–1030. doi: 10.1007/s11277-014-1915-8

[2] Li L, Chigan C. Fuzzy C-means clustering based secure fusion strategy in collaborative spectrum sensing. IEEE International Conference on Communication. 2014:1355–1360.

[3] Sesham S, Sabat SL. Spectrum sensing for cognitive radio networks White Space Communication, Springer International Publishing 2015:117–151.

[4] Balaji V, Hota C. Efficient cooperative spectrum sensing in cognitive radio using coalitional game model. IEEE International Conference on Contemporary Computing and Informatics. 2015:899–907.

[5] He X, Dai H, Nin P. A Byzantine attack defender in cognitive radio networks: the conditional frequency check. IEEE Transactions on Wireless Communications. 2013;12(5):2512–2523. doi: 10.1109/TWC.2013.031313.121551

[6] Chen R, Park JMJ, Bian K. Robustness against Byzantine failures in distributed spectrum sensing. Computer Communications. 2012;35(17):2115–2124. doi: 10.1016/j.comcom.2012.07.014

[7] Chen CY, Chou YH, Chou HC, Lo HC. Secure centralized spectrum sensing for cognitive radio networks. Wireless Networks. 2012;18(6):667–677. doi: 10.1007/s11276-012-0426-3

[8] Lu J, Wei P. Improved cooperative spectrum sensing based on the reputation in cognitive radio networks. International Journal of Electronics. 2015;102(5):855–863. doi: 10.1080/00207217.2014.942887

[9] Sharifi AA, Niya JM. Securing collaborative spectrum sensing against malicious attackers in cognitive radio networks. Wireless Personal Communications. 2016;90(1):1–17. doi: 10.1007/s11277-016-3331-8

[10] Alizadeh H, Sharifi AA, Niya JM, Seyedarabi H. Attack-aware cooperative spectrum sensing in cognitive radio networks under Byzantine attack. Journal of Communication Engineering. 2017;6(1):81–98.

[11] Wang M, Liu B, Zhang C. Detection of collaborative SSDF attacks using abnormality detection algorithm in cognitive radio networks. IEEE International Conference on Communications Workshops. 2013:342–346.

[12] Zeng K, Peng QH, Tang YX. Mitigating spectrum sensing data falsification attacks in hard-decision combining cooperative spectrum sensing. Science China Information Sciences. 2014;57(4):1–9. doi: 10.1007/s11432-013-4847-0

[13] Sharifi AA, Sharifi M, Niya JM. Reputation-based likelihood ratio test with anchor nodes assistance. 8th International Symposium on Telecommunications (IST). 2016:51–56.

[14] Ye F, Zhang X, Li Y, Tang C. Faithworthy collaborative spectrum sensing based on credibility and evidence theory for cognitive radio networks. Symmetry. 2017;9(3):36 doi: 10.3390/sym9030036

[15] Lixiang L, Jürgen K, Yixian Y, Guole L. Prevention and trust evaluation scheme based on interpersonal relationships for large-scale peer-to-peer networks. Mathematical Problems in Engineering. 2014.

[16] Wang X, Jia M, Guo Q, Gu X, Zhang G. Reputation-based cooperative spectrum sensing algorithm for mobile cognitive radio networks. China Communications. 2017;14(1):124–134. doi: 10.1109/CC.2017.7839763

[17] Jana S, Zeng K, Cheng W, Mohapatra P. Trusted collaborative spectrum sensing for mobile cognitive radio networks. IEEE Transactions on Information Forensics and Security. 2013;8(9):1497–1507. doi: 10.1109/TIFS.2013.2273305

[18] Dong X, Haipeng P, Lixiang L, Yixian Y. Efficient post-quantum secure network coding signatures in the standard model. KSII Transactions on Internet and Information Systems (TIIS). 2016 5;10(1):2427–2445.

[19] Dong X, Haipeng P, Lixiang L, Yixian Y. An efficient privacy-preserving scheme for secure network coding based on compressed sensing. AEU-International Journal of Electronics and Communications. 2017;71(1):33–42.

[20] Amin T. Performance analysis of secondary users in heterogeneous cognitive radio network. Spring 2016.

[21] Madbushi S, Raut R, Rukmini MSS. Security issues in cognitive radio: a review. Microelectronics, Electromagnetics and Telecommunications. 2016:121–134.

[22] Zhao Y, Paul P, Xin CS, Song M. Performance analysis of spectrum sensing with mobile SUs in cognitive radio networks. IEEE International Conference on Communications. 2014:2761–2766.

[23] Goldsmith A. Wireless communication. Cambridge. U.K.: Cambridge Univ. Press 2006.

[24] Nadendla YSS, Han YS, Varshney PK. Distributed inference with M-ary quantized data in the presence of Byzantine attacks. IEEE Transactions on Signal Processing. 2014;62(10):2681–2695. doi: 10.1109/TSP.2014.2314072

[25] Chen H, Zhou M, Xie L, Li J. Cooperative spectrum sensing with M-ary quantized data in cognitive radio networks under SSDF attacks. IEEE Transactions on Wireless Communications. 2017;16(8):5244–5257. doi: 10.1109/TWC.2017.2707407

[26] Sucasas V, Althunibat S, Radwan A, Marques H, Rodriguez J, Vahid S, et al Lightweight security against combined IE and SSDF attacks in cooperative spectrum sensing for cognitive radio networks. Security and Communication Networks. 2015;8(18):3978–3994. doi: 10.1002/sec.1315

[27] Zhang L, Ding G, Wu Q, Zou Y, Han Z, Wang J. Byzantine attack and defense in cognitive radio networks: a survey. IEEE Communications Surveys & Tutorials, 2015;17(3):1342–1363. doi: 10.1109/COMST.2015.2422735

[28] Sharifi AA, Niya MJM. Defense against SSDF attack in cognitive radio networks: attack-aware collaborative spectrum sensing approach. IEEE Communications Letters. 2016;20(1):93–96. doi: 10.1109/LCOMM.2015.2499286

[29] Rawat AS, Anand P, Chen H, Varshney PK. Collaborative spectrum sensing in the presence of Byzantine attacks in cognitive radio networks. IEEE Transactions on Signal Processing. 2011 2;59(2):774–786. doi: 10.1109/TSP.2010.2091277

[30] Vegi MK. Study of Byzantine attacks and countermeasures in spectrum sensing. Oklahoma State University 2014.

[31] Kailkhura B, Han YS, Brahma S, Varshney PK. Distributed Bayesian detection in the presence of Byzantine data. IEEE Transactions on Signal Processing. 2015;63(19):5250–5263. doi: 10.1109/TSP.2015.2450191

[32] Varshney PK. Distributed detection and data fusion. New York, NY, USA: Springer-Verlag 1996.

[33] Clement JC. Jettison the defectives: a robust cooperative spectrum sensing scheme in a cognitive radio network. Circuits Systems & Signal Processing. 2017;1:1–21.

[34] Althunibat S, Denise BJ, Granelli F. Identification and punishment policies for spectrum sensing data falsification attackers using delivery-based assessment. IEEE Transactions on Vehicular Technology. 2016;65(9):7308–7321. doi: 10.1109/TVT.2015.2497349

[35] Nguyen-Thanh N, Koo I. An efficient ordered sequential cooperative spectrum sensing scheme based on evidence theory in cognitive radio,. IEICE transactions on communications. 2010;93(12):3248–3257. doi: 10.1587/transcom.E93.B.3248

[36] Rappaport TS. Wireless communications: principles and practice. New Jersey: prentice hall PTR, 1996:2.

