# Supplementary figures and images for: Sequential cooperative spectrum sensing in the presence of dynamic Byzantine attack for mobile networks
Source: PLoS One. 2018 Jul 5;13(7):e0199546. doi: 10.1371/journal.pone.0199546 (PMC6033420; doi:10.1371/journal.pone.0199546)

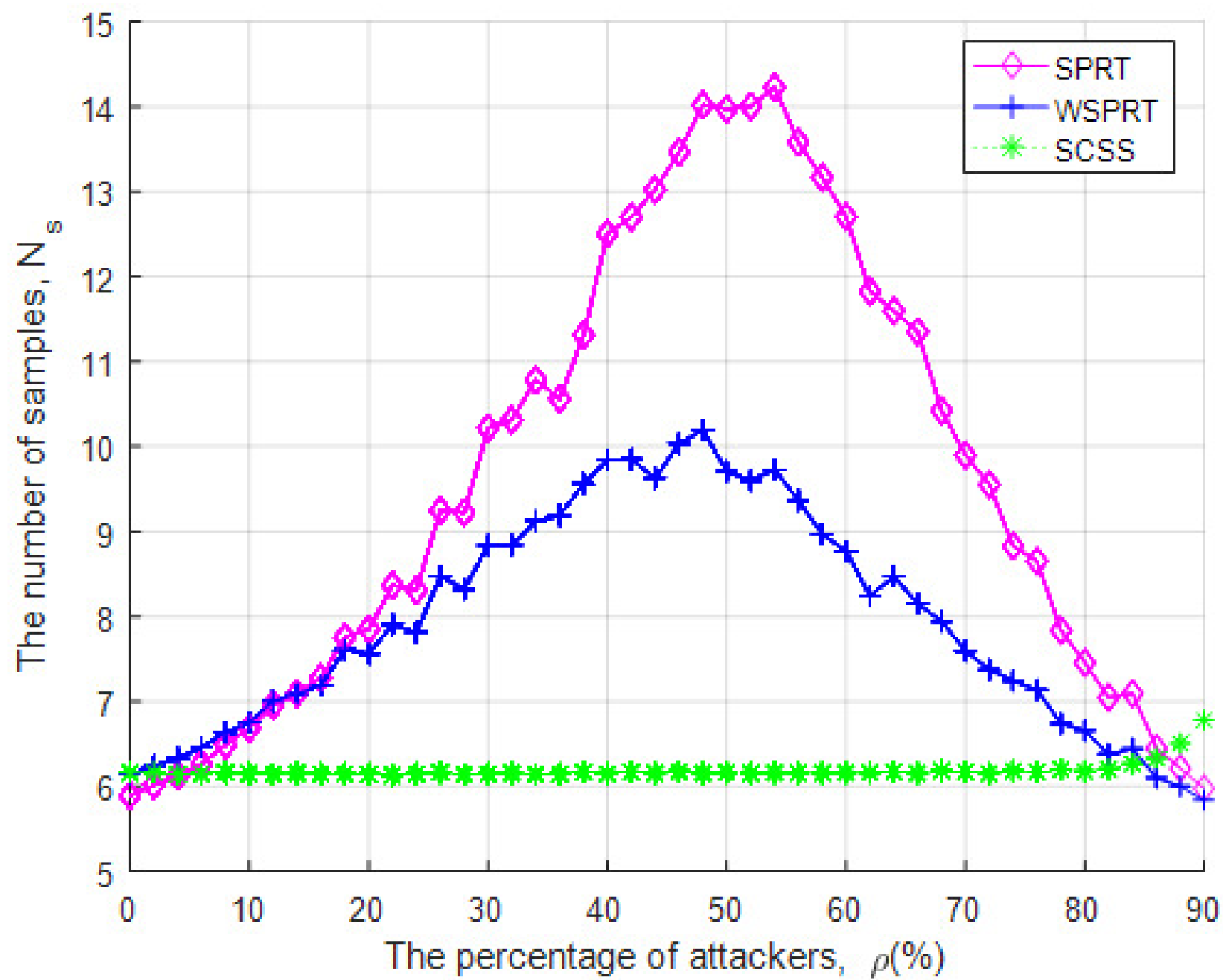

Supplement: S1 Fig — (ZIP) [file pone.0199546.s001.zip › S1B_Fig.pdf]

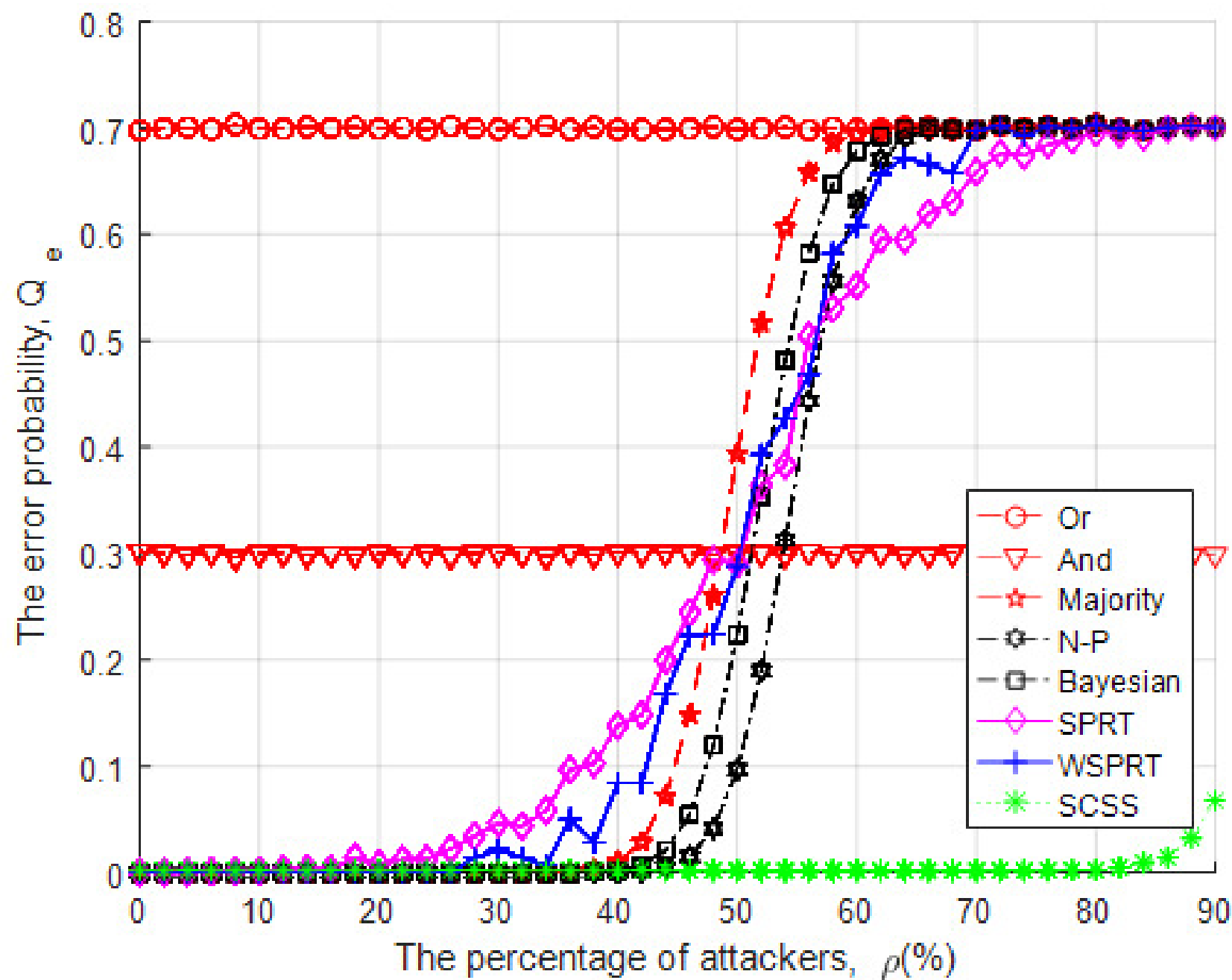

Supplement: S1 Fig — (ZIP) [file pone.0199546.s001.zip › S1A_Fig.pdf]

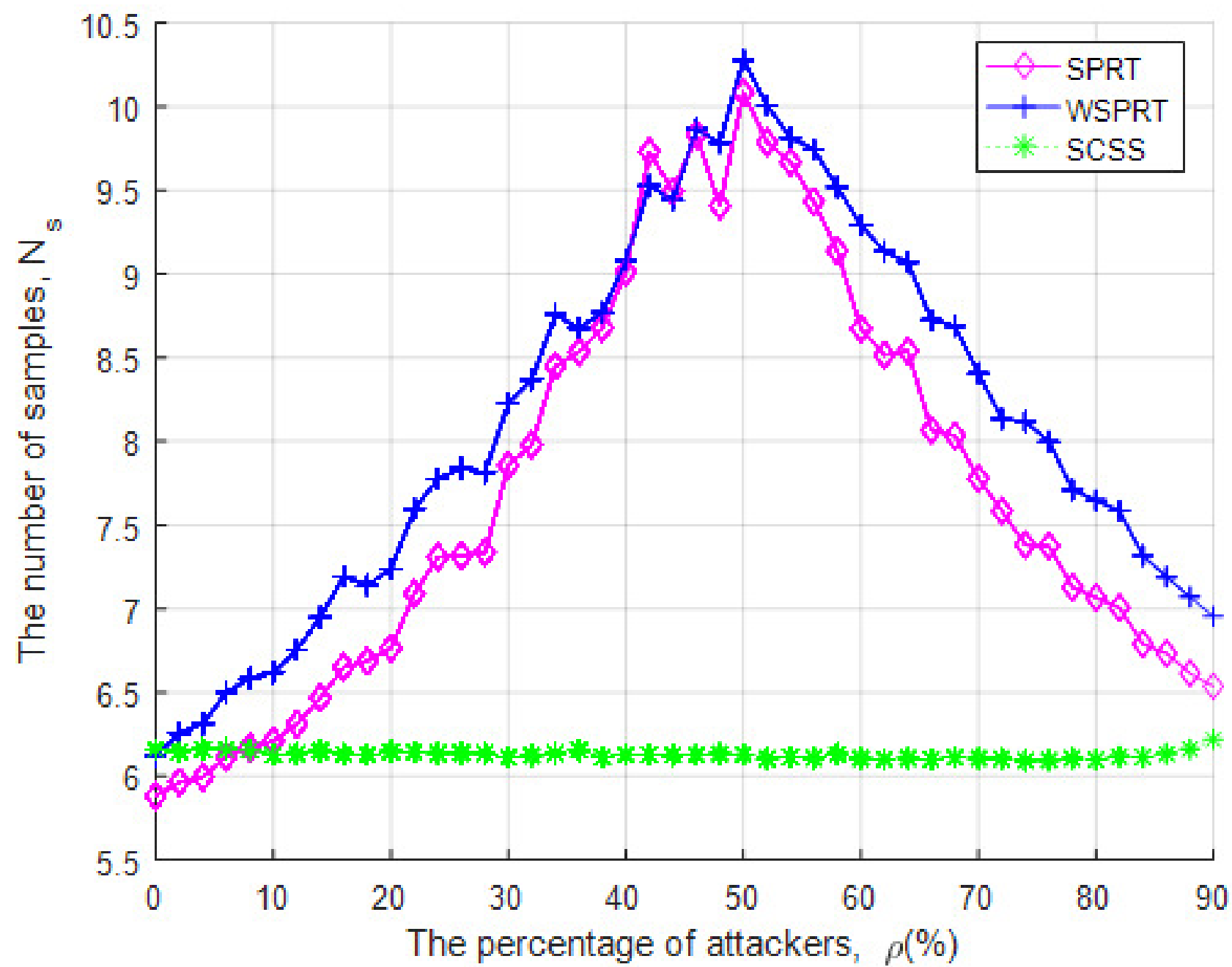

Supplement: S2 Fig — (ZIP) [file pone.0199546.s002.zip › S2B_Fig.pdf]

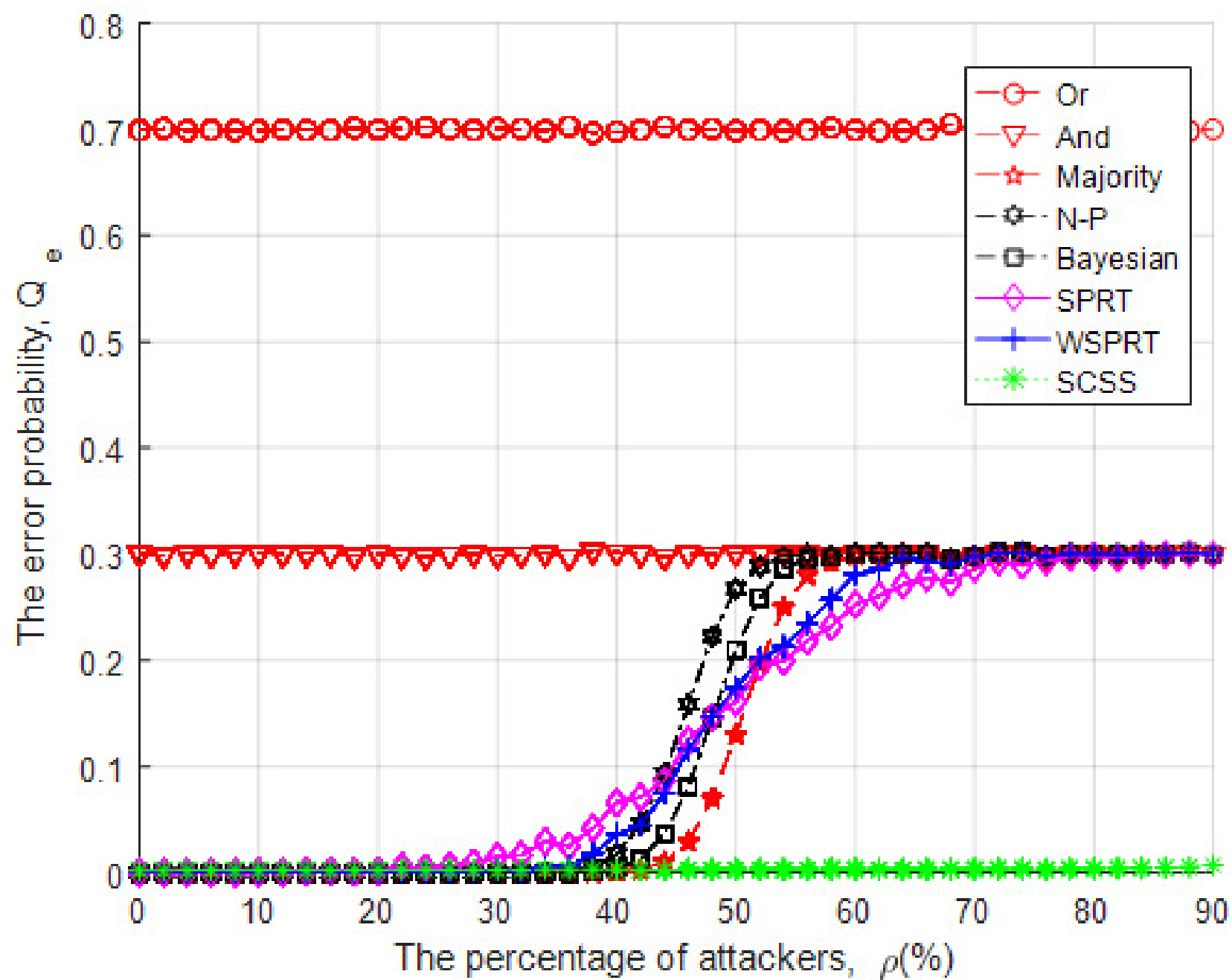

Supplement: S2 Fig — (ZIP) [file pone.0199546.s002.zip › S2A_Fig.pdf]

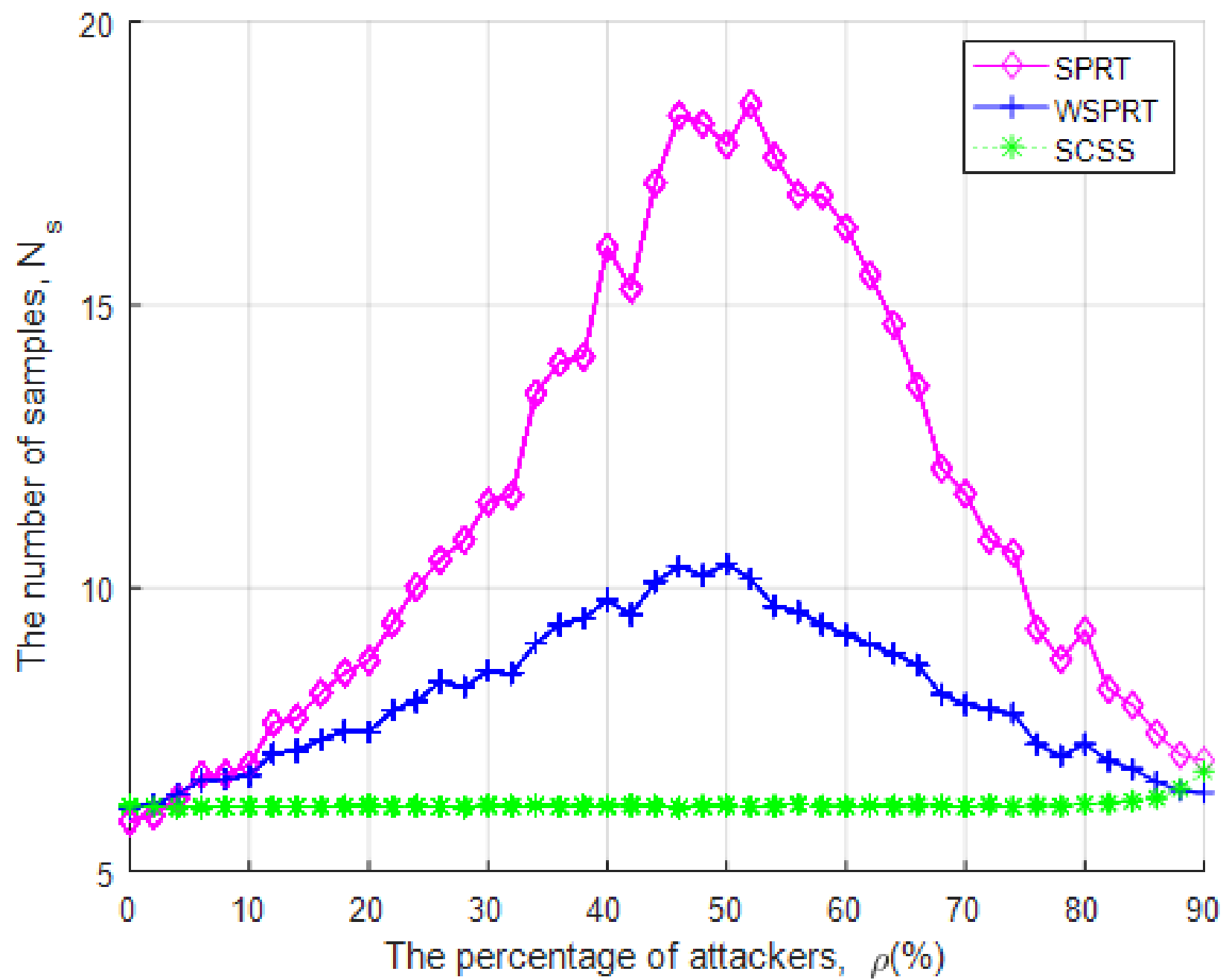

Supplement: S3 Fig — (ZIP) [file pone.0199546.s003.zip › S3B_Fig.pdf]

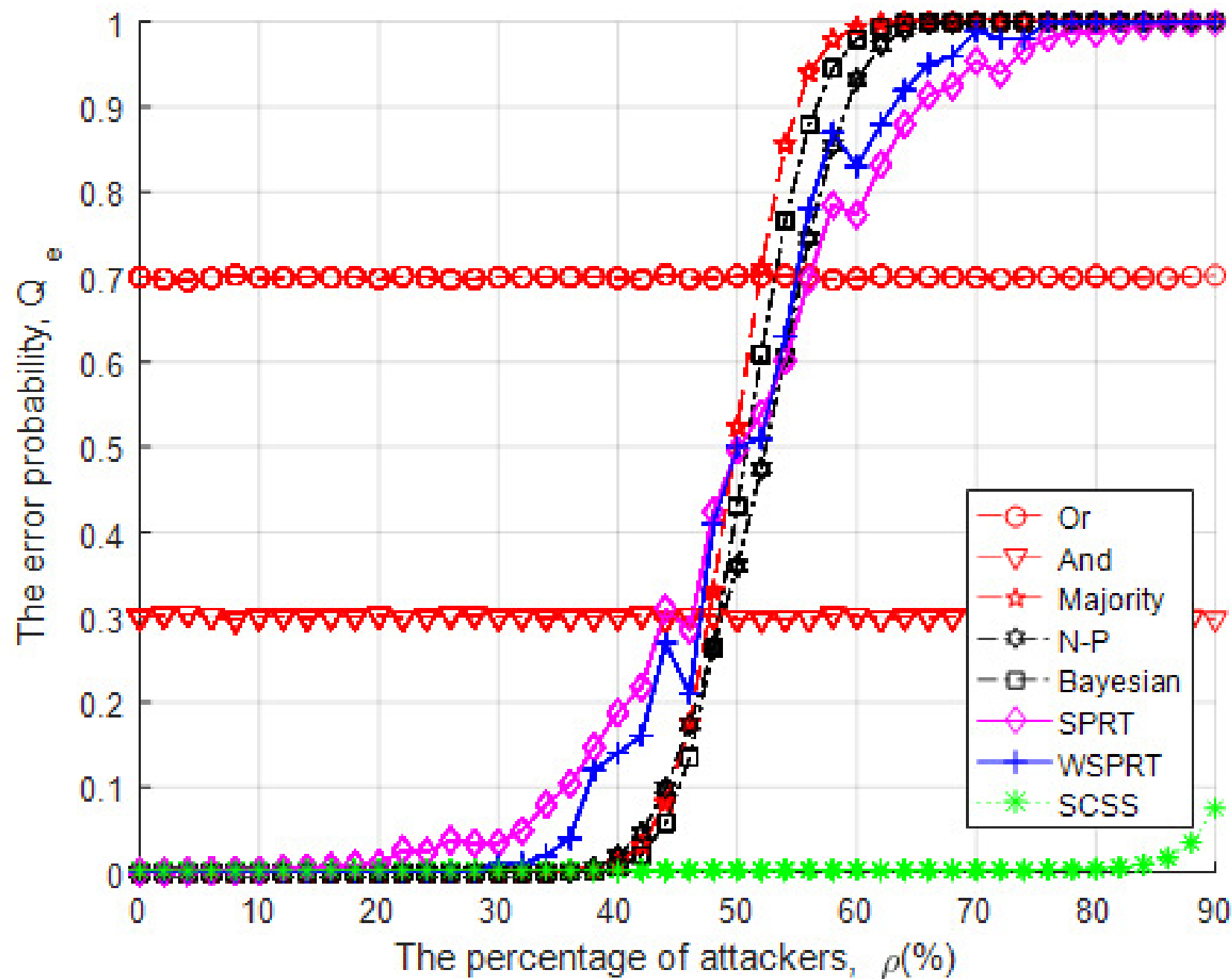

Supplement: S3 Fig — (ZIP) [file pone.0199546.s003.zip › S3A_Fig.pdf]

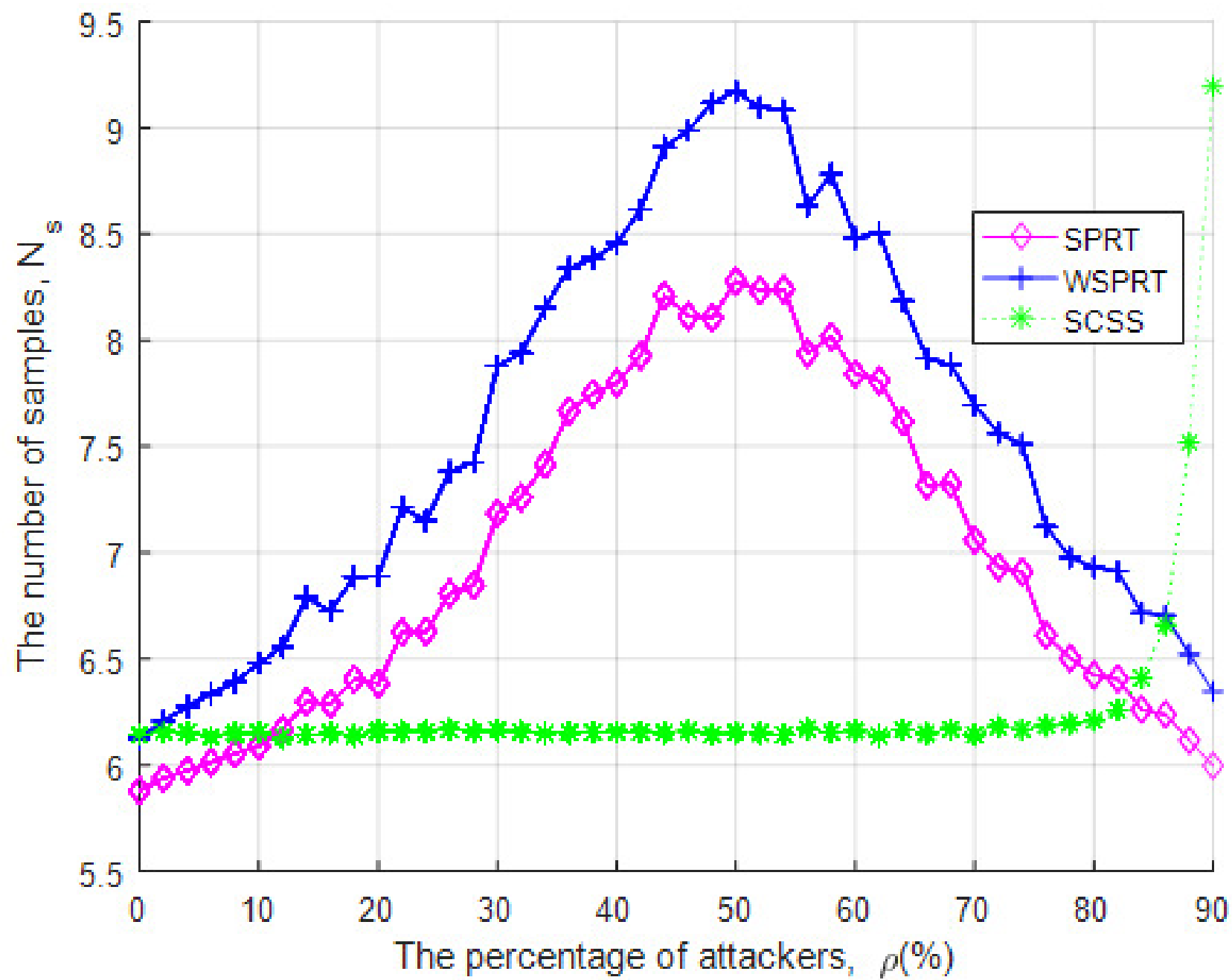

Supplement: S4 Fig — (ZIP) [file pone.0199546.s004.zip › S4B_Fig.pdf]

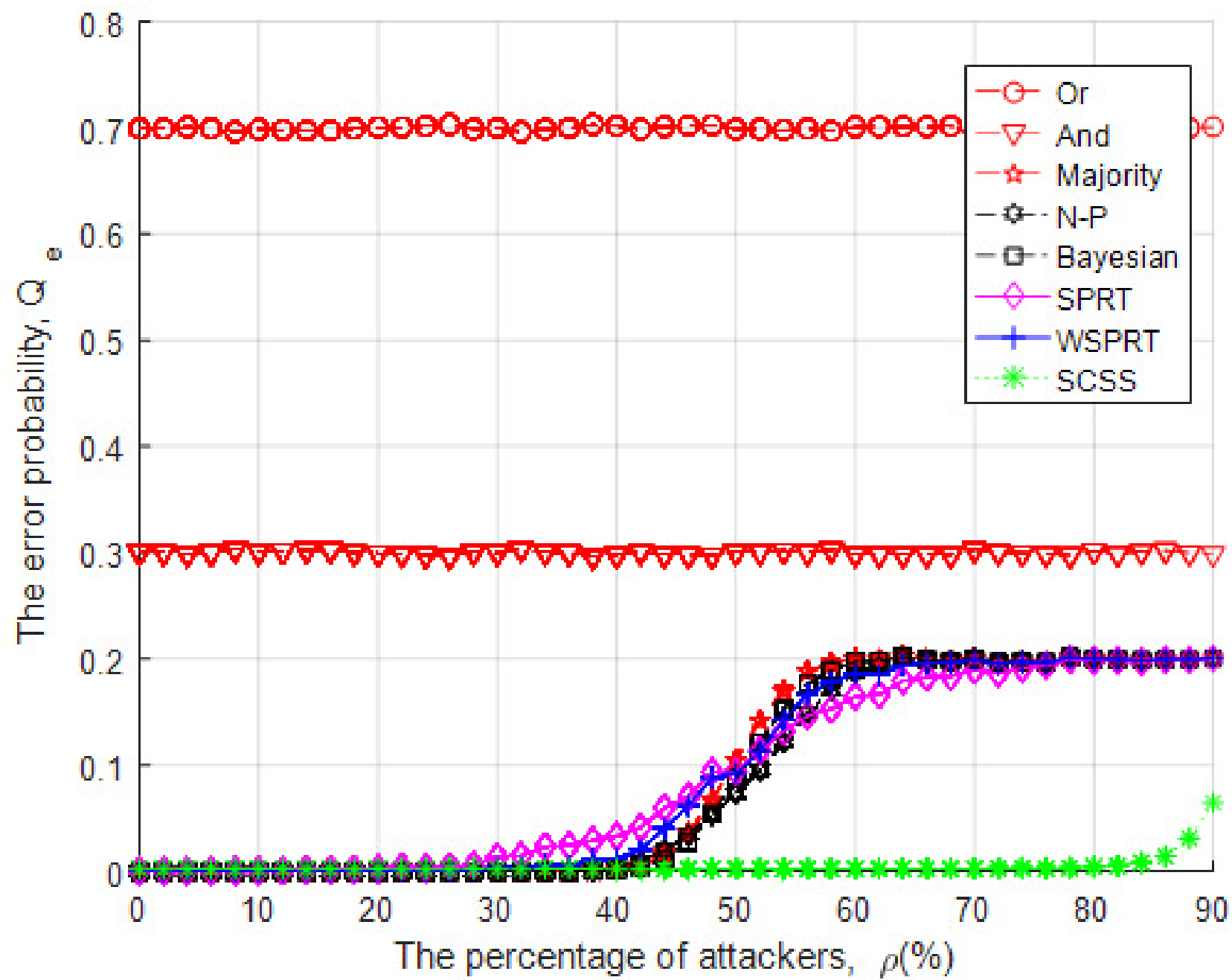

Supplement: S4 Fig — (ZIP) [file pone.0199546.s004.zip › S4A_Fig.pdf]

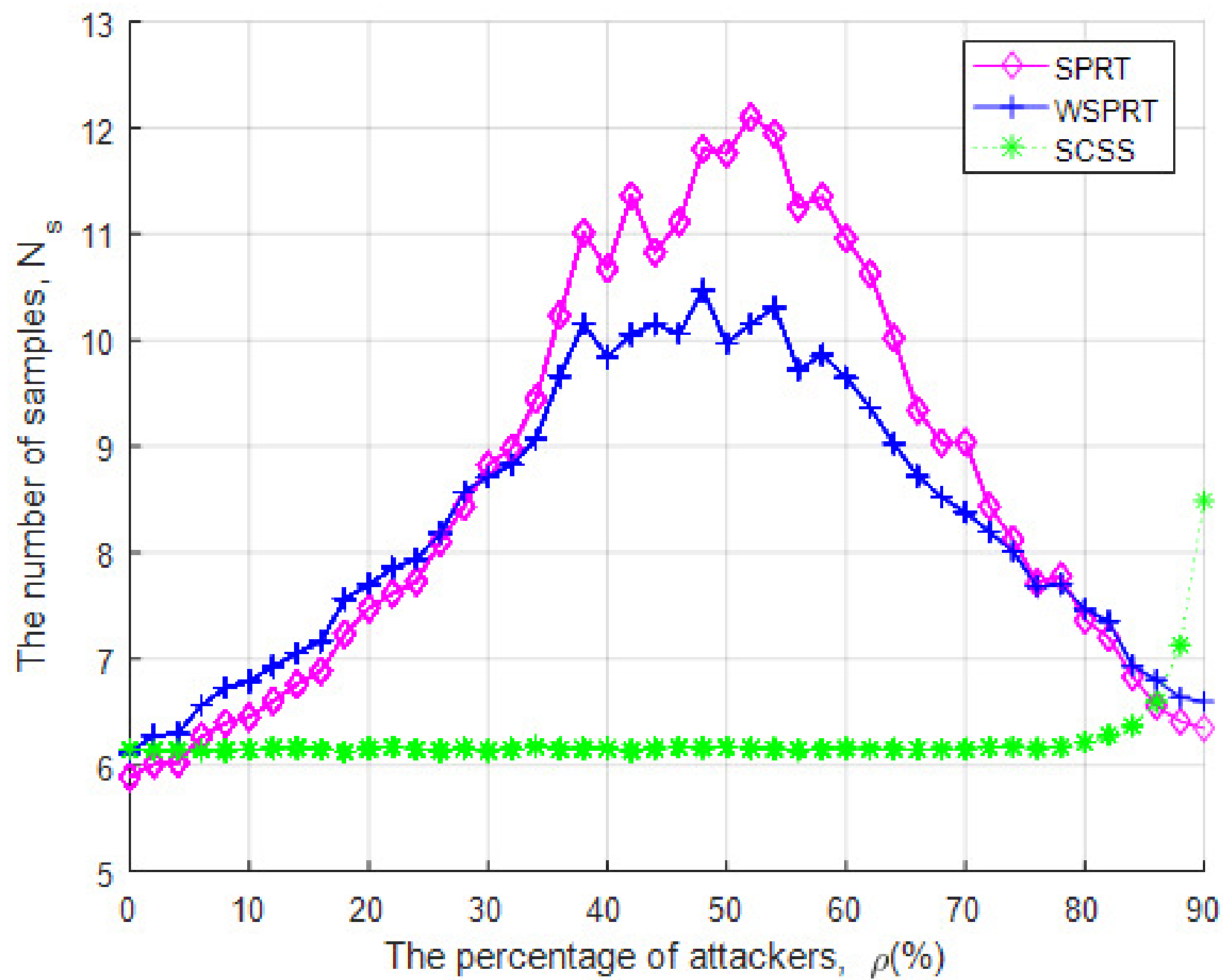

Supplement: S5 Fig — (ZIP) [file pone.0199546.s005.zip › S5B_Fig.pdf]

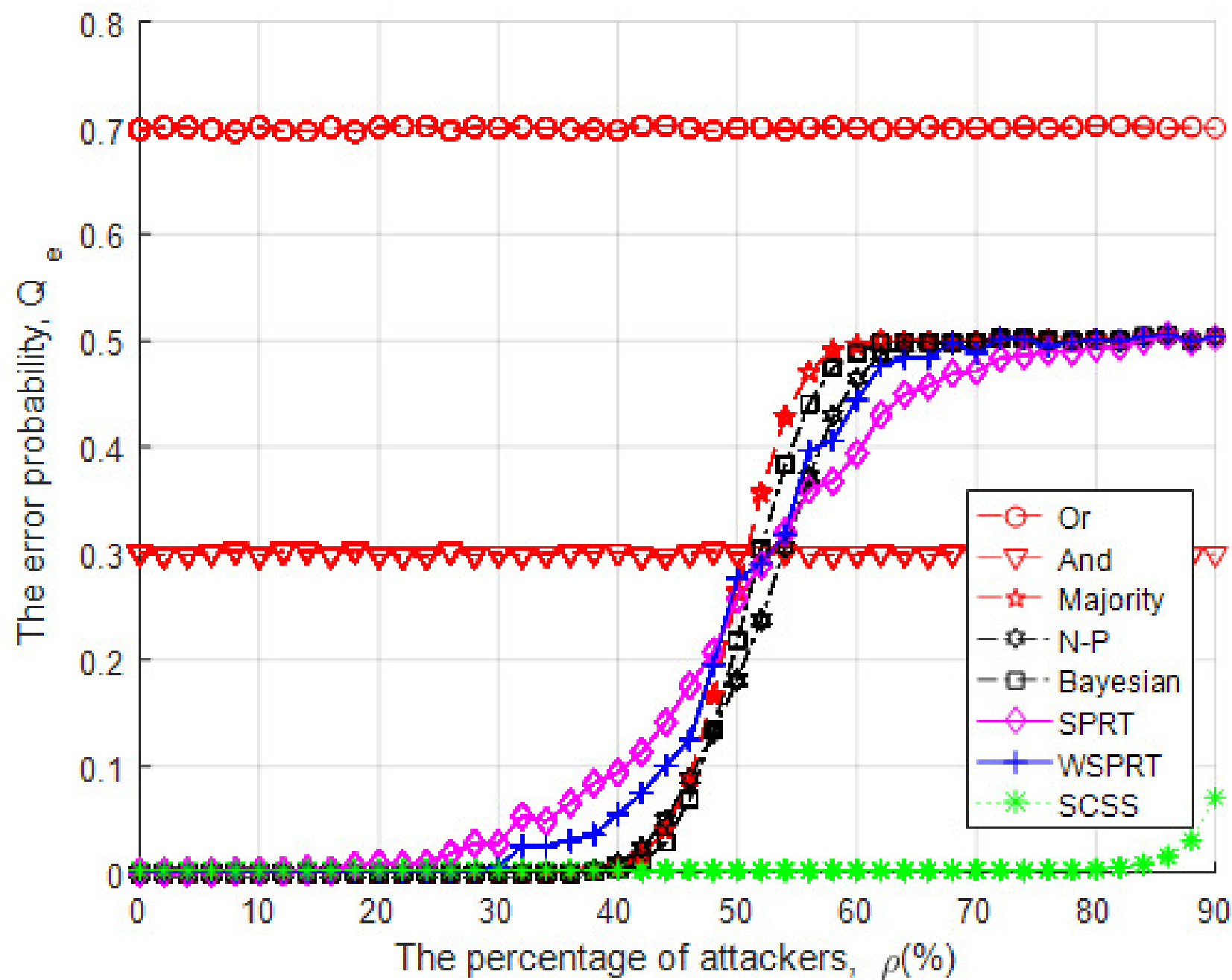

Supplement: S5 Fig — (ZIP) [file pone.0199546.s005.zip › S5A_Fig.pdf]

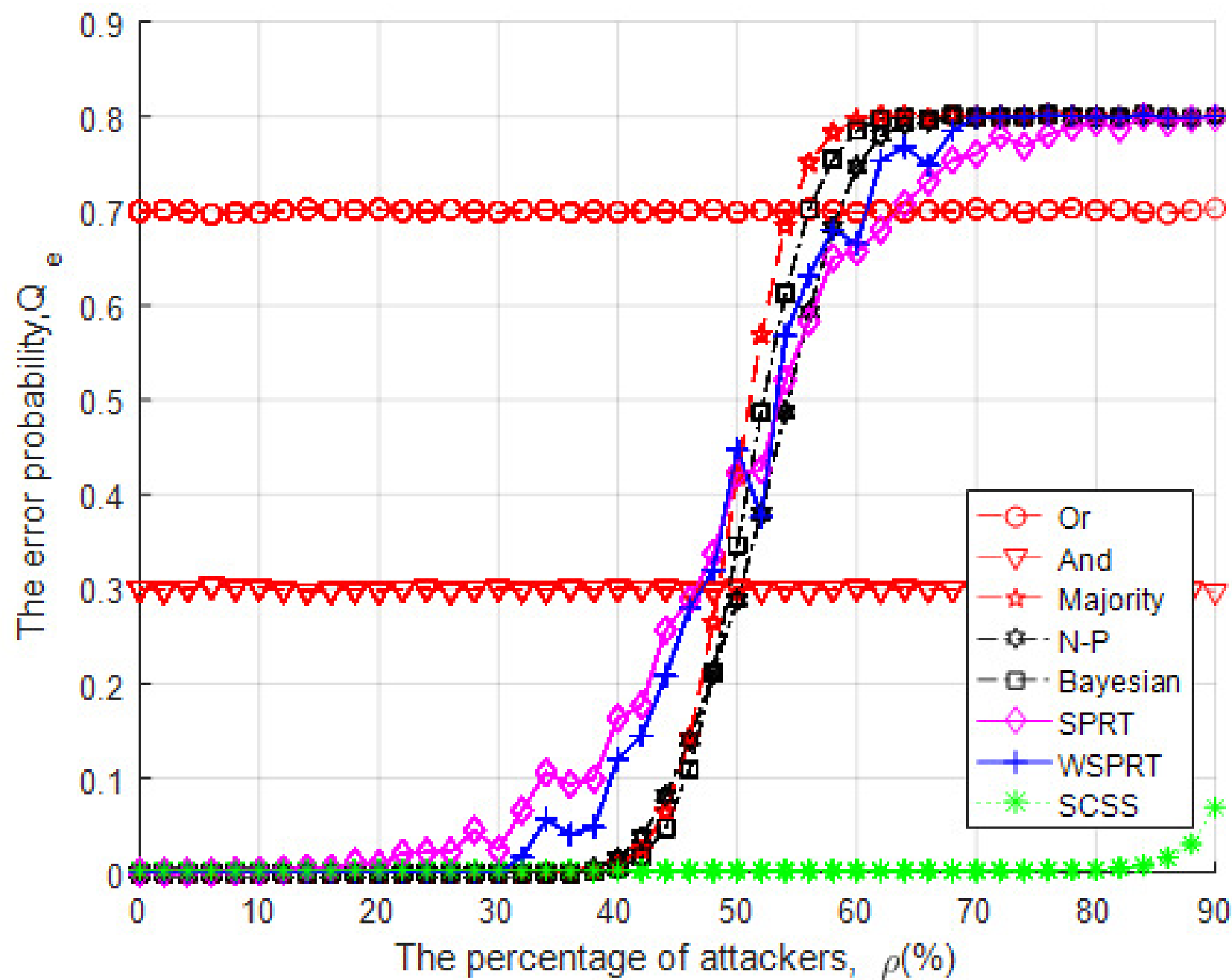

Supplement: S6 Fig — (ZIP) [file pone.0199546.s006.zip › S6A_Fig.pdf]

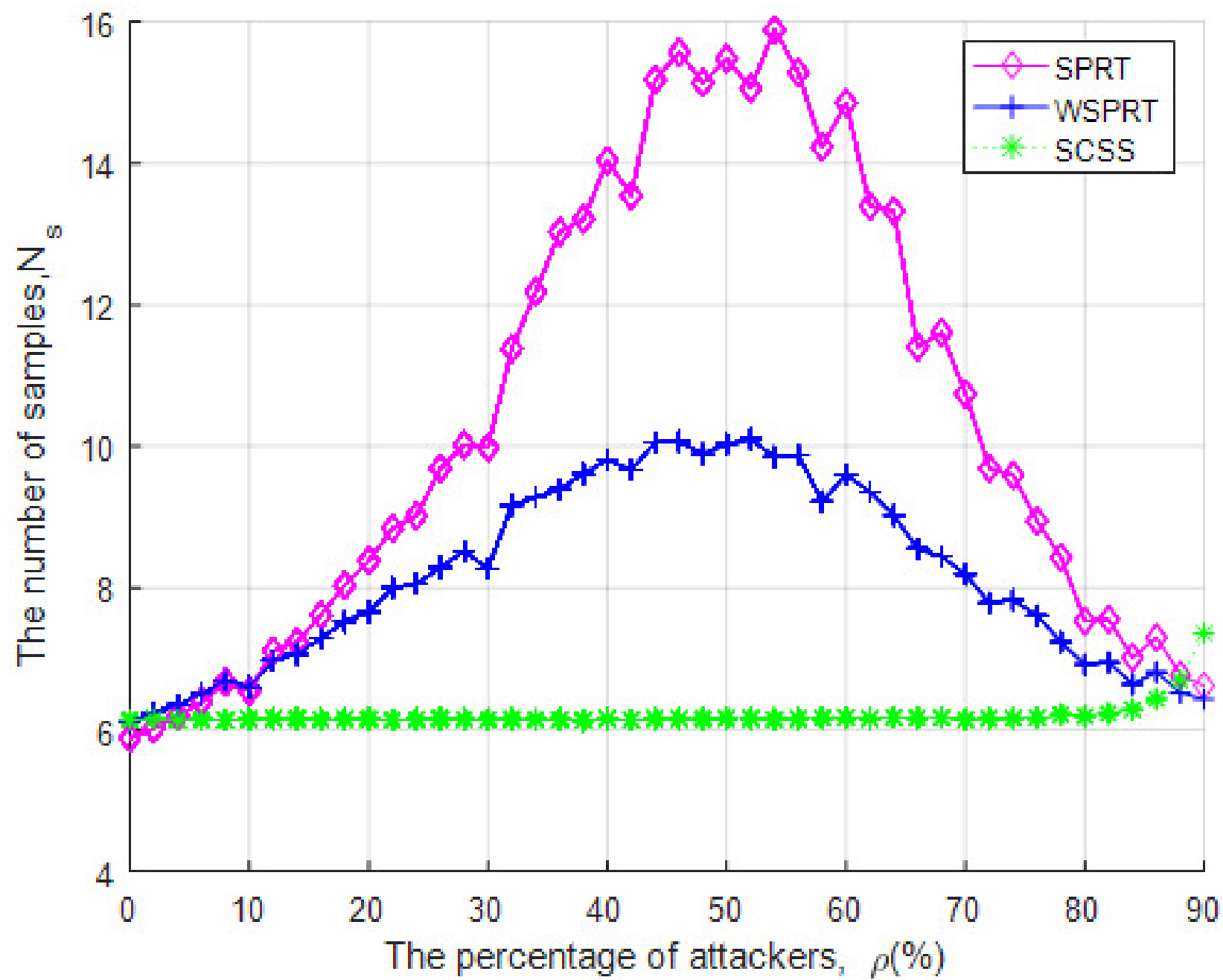

Supplement: S6 Fig — (ZIP) [file pone.0199546.s006.zip › S6B_Fig.pdf]
